# Phosphorylated p-70S6K predicts tamoxifen resistance in postmenopausal breast cancer patients randomized between adjuvant tamoxifen versus no systemic treatment

**DOI:** 10.1186/bcr3598

**Published:** 2014-01-21

**Authors:** Karin Beelen, Mark Opdam, Tesa M Severson, Rutger HT Koornstra, Andrew D Vincent, Jelle Wesseling, Jettie J Muris, Els MJJ Berns, Jan B Vermorken, Paul J van Diest, Sabine C Linn

**Affiliations:** 1 Department of Molecular Pathology, The Netherlands Cancer Institute, Plesmanlaan 121, Amsterdam 1066 CX, the Netherlands; 2 Department of Biometrics, The Netherlands Cancer Institute, Amsterdam, the Netherlands; 3 Department of Pathology, The Netherlands Cancer Institute, 's-Gravendijkwal 230, Amsterdam 3015 CE, the Netherlands; 4 Department of Medical Oncology, Erasmus University Medical Center-Cancer Center, Rotterdam, the Netherlands; 5 Department of Medical Oncology, Antwerp University Hospital, Wilrijkstraat 10, Edegem 2650, Belgium; 6 Department of Pathology, University Medical Center Utrecht, Heidelberglaan 100, Utrecht 3584 CX, the Netherlands; 7 Department of Medical Oncology, The Netherlands Cancer Institute, Amsterdam, the Netherlands

## Abstract

**Introduction:**

Activation of the phosphatidylinositol-3-kinase (PI3K) and/or mitogen-activated protein kinase (MAPK) pathways results in anti-estrogen resistance *in vitro*, but a biomarker with clinical validity to predict intrinsic resistance has not been identified. In metastatic breast cancer patients with previous exposure to endocrine therapy, the addition of a mammalian target of rapamycine (mTOR) inhibitor has been shown to be beneficial. Whether or not patients on adjuvant endocrine treatment might benefit from these drugs is currently unclear. A biomarker that predicts intrinsic resistance could potentially be used as companion diagnostic in this setting. We tested the clinical validity of different downstream-activated proteins in the PI3K and/or MAPK pathways to predict intrinsic tamoxifen resistance in postmenopausal primary breast cancer patients.

**Methods:**

We recollected primary tumor tissue from patients who participated in a randomized trial of adjuvant tamoxifen (1–3 years) versus observation. After constructing a tissue micro-array, cores from 563 estrogen receptor α positive were immunostained for p-AKT(Thr308), p-AKT(Ser473), p-mTOR, p-p706SK and p-ERK1/2. Cox proportional hazard models for recurrence free interval were used to assess hazard ratios and interactions between these markers and tamoxifen treatment efficacy.

**Results:**

Interactions were identified between tamoxifen and p-AKT(Thr308), p-mTOR, p-p70S6K and p-ERK1/2. Applying a conservative level of significance, p-p70S6K remained significantly associated with tamoxifen resistance. Patients with p-p70S6K negative tumors derived significant benefit from tamoxifen (HR 0.24, *P* < 0.0001), while patients whose tumor did express p-p70S6K did not (HR = 1.02, *P* =0.95), *P* for interaction 0.004. In systemically untreated breast cancer patients, p-p70S6K was associated with a decreased risk for recurrence.

**Conclusions:**

Patients whose tumor expresses p-p70S6K, as a marker of downstream PI3K and/or MAPK pathway activation, have a favorable prognosis, but do not benefit from adjuvant tamoxifen. A potential benefit from inhibitors of the PI3K/Akt/mTOR pathway in these patients needs to be further explored.

## Introduction

Activation of the mitogen-activated protein kinase (MAPK) and phosphatidylinositol-3-kinase (PI3K) pathways confers anti-estrogen resistance *in vitro*[1-3]. Apart from activation by the canonical pathway drivers (*PIK3CA* mutations [4], loss of PTEN [5] or overexpression of growth factor receptors such as human epidermal growth factor receptor 2 (HER2) [6] and insulin like growth factor 1 receptor [7]), *in vitro* data have shown that the PI3K pathway can also be activated in response to estrogen depletion. This results in acquired hormone-resistant breast cancer cells that are sensitive to PI3K/mammalian target of rapamycine (mTOR) inhibition [8]. These preclinical data support the clinical observation that estrogen receptor alpha (ERα)-positive metastatic breast cancer patients with prior exposure to aromatase inhibitors derive substantial benefit from the addition of an mTOR inhibitor [9]. Whether or not patients who are primarily resistant to adjuvant endocrine therapy might benefit from PI3K and/or MAPK pathway inhibition remains to be defined. A biomarker of an activated PI3K and/or MAPK pathway with clinical validity to predict resistance in the adjuvant setting has not been identified [10], but could potentially be used as a companion diagnostic for non-ERα-targeted drugs, such as an mTOR inhibitor.

Several canonical pathway drivers, such as *PIK3CA* mutations [11,12], loss of PTEN [12], and HER2 [13], have been studied for their validity to predict resistance. However, none of these drivers significantly predicts lack of benefit from endocrine therapy. An important issue to be considered is that the presence of these drivers in clinical samples may not necessarily result in high activation of downstream proteins [12,14]. *In vitro*, PI3K pathway activation leads to phosphorylation of AKT and subsequently of mTOR and p70S6K [10]. Phosphorylation of extracellular signal-regulated kinase (ERK)1/2 is a result of MAPK pathway activation [15] and mediates activation of p70S6K [16]. However, relatively moderate activation of the PI3K pathway was seen in tumors with a *PIK3CA* exon 20 mutation-associated gene signature [14]. In addition, in a large series of primary breast cancer tumors, reverse-phase protein analysis did not show activation of the typical downstream proteins in the PI3K pathway in PIK3CA mutated luminal A tumors [17]. The activation status of downstream proteins rather than the presence or absence of a canonical driver therefore probably ultimately defines anti-estrogen sensitivity in breast cancer patients.

We hypothesized that activated proteins downstream in the PI3K and/or MAPK kinase pathways could potentially be used as a marker that separates patients who are likely to benefit from adjuvant tamoxifen treatment from those who are primarily resistant to this drug. The aim of our study was therefore to investigate the predictive value of different downstream activated proteins in the PI3K and/or MAPK pathways in a large series of ERα-positive postmenopausal breast cancer patients randomized between adjuvant tamoxifen versus no systemic treatment.

## Methods

### Patients and materials

We have recollected primary tumor tissue blocks from stage I to III postmenopausal breast cancer patients who were randomized (2:1) between 1 year of tamoxifen (30 mg/day) versus no adjuvant therapy (IKA trial, 1982 to 1994) [18,19]. Study data were part of the Oxford meta-analysis [20]. After 1989, based on two interim analyses showing a significant improvement in recurrence-free survival in lymph node-positive patients, node-positive patients in this trial skipped the first randomization and all received 1 year of tamoxifen. After 1 year a second randomization was performed to receive another 2 years of tamoxifen or to stop further treatment. In total, 1,662 patients were included. None of these patients received adjuvant chemotherapy. The patient characteristics and clinical outcome of the original study group (1,662 patients) have been presented elsewhere [19].

Sufficient tumor material was available for 739 patients, who did not differ in prognostic factors from the total group (Table S1 in Additional file 1). After revision of ERα status as assessed with immunohistochemistry (IHC), a total of 563 ERα-positive tumors were used for subsequent analysis. We used a cutoff value ≥10% of positive tumor cells for ERα positivity, since this is common practice in the Netherlands and also this would avoid the potential inclusion of basal-like tumors [21] in our analysis. The original trial was approved by the central ethics committee of the Netherlands Cancer Institute and informed consent was obtained from all study participants. For this retrospective translational study, no additional consent was required according to Dutch legislation [22] since the use of archival pathology left-over material does not interfere with patient care. Tumor tissue was handled according to the Dutch code of conduct for dealing responsibly with human tissue in the context of health research [23].

### Immunohistochemistry

Tissue microarrays (TMAs) were constructed using formalin-fixed paraffin-embedded tumor blocks. A total of three (0.6 mm) cores per tumor were embedded in the TMAs that were stained for ERα, progesterone receptor (PgR) and HER2. ERα and PgR were considered positive when ≥10% of invasive cells showed nuclear reactivity. HER2 was considered positive when membranous staining was DAKO score 3 [24]. In the case of DAKO score 2, chromogenic *in situ* hybridization was performed. For tumors without sufficient cores in the TMA, whole slides were cut and assessed for ERα (*n* = 60), PgR (*n* = 55) and HER2 (*n* = 36). The tumor grade was scored on a hematoxylin and eosin-stained slide using the modified Bloom–Richardson score [25].

Antibodies used for immunohistochemistry of downstream phosphorylated (p) proteins are shown in Table S2 in Additional file 1. For p-AKT(Ser473), antigen retrieval was performed using citrate buffer and slides were incubated overnight with antibody (dilution 1:50). All other phospho-protein stainings (p-AKT(Thr308), p-mTOR, p-ERK1/2 and p-p70S6K) were performed using a standardized protocol on the Ventana Benchmark® Ultra system (Ventana Medical Systems, Tucson, USA). To ensure phospho-specificity of the antibodies, for each antibody a test TMA containing positive cores was dephosphorylated by λ-phosphatase before staining, resulting in disappearance of the positive staining (Figure S1 in Additional file 1).

Cytoplasmic intensity (0 to 3) was assessed for p-AKT(Ser473), p-AKT(Thr308) and p-p70S6K. The percentage of tumor cells with submembranous staining was scored for p-mTOR, and the proportion of positive nuclei was scored for p-ERK1/2. For each staining, one of the TMAs was quantified independently in a blinded manner by a second observer to calculate inter-observer variability. For further analyses, we used the scores produced by the first observer (MO).

Since the stability of phospho-proteins is a matter of debate [26], we tested whether the relative age of tumor samples (divided in quartiles) was associated with quantitative phospho-protein staining for each of these markers. In addition, to test for a possible effect of different fixation procedures, we tested whether the inclusion center was associated with differences in quantitative phospho-protein expression.

### Statistical analysis

The recurrence-free interval was defined as the time from the date of first randomization until the occurrence of a local, regional or distant recurrence or breast cancer-specific death [27]. Since a secondary contralateral breast tumor cannot be inferred from the molecular make-up of the primary tumor, while the other type of events can be inferred in relation to tamoxifen resistance of the primary tumor, this was not considered an event and these patients were censored at the date of this occurrence. The association between expression of downstream activated proteins and known prognostic factors was tested using Fisher’s exact test.

We hypothesized that high expression of downstream activated proteins is associated with tamoxifen resistance. Our primary analysis was therefore to test whether tamoxifen benefit was dependent on any of the downstream activated proteins in the PI3K and/or MAPK pathway. We analyzed these markers as a binary factor, using the median level as the cutoff value. Adjusted Cox proportional hazard regression analyses were performed including an interaction variable. Covariates included age (≥65 vs. <65), grade (grade 3 vs. grade 1 to 2), tumor size (T3 to T4 vs. T1 to T2), HER2 status (positive vs. negative), and PgR status (positive vs. negative). All survival analyses were stratified for nodal status. Owing to the multiple co-primary endpoints of this study, we apply a more conservative level of significance (α = 0.01) when assessing the interactions.

Further exploratory analyses examined tamoxifen benefit when the markers were implemented as continuous linear variables. For those continuous linear variables that showed an interaction with tamoxifen treatment, we explored which level of dichotomization best predicted tamoxifen benefit, by comparing Akaike’s information criteria of the Cox proportional hazards models for all possible cutoff values. In addition, based on knowledge derived from preclinical studies [1,3], we explored whether a composed variable of either high p-ERK1/2 or high p-mTOR – indicating the activation of either the MAPK pathway or the PI3K pathway – was associated with tamoxifen resistance. To assess the prognostic value of the phospho-proteins, we analyzed their putative prognostic potential in the subgroup of patients who were randomized to the control arm. The reason why we did not use all patients and corrected for tamoxifen treatment is that this correction would assume that all ERα-positive breast cancer patients would derive similar benefit from tamoxifen. Since the phospho-protein might be associated with tamoxifen resistance, simply correcting for the assumed tamoxifen benefit without a correction for a potential interaction between treatment and phospho-protein could bias the analysis for prognostic potential. Survival curves were constructed using the Kaplan–Meier method. This study complied with reporting recommendations for tumor marker prognostic studies (REMARK) criteria [28] as outlined in Table S3 in Additional file 1.

## Results

### Association of downstream activated proteins in the PI3K and/or MAPK pathways with clinico-pathological factors

The inter-observer variability analyzed using the (weighted) Cohen’s kappa coefficient is depicted in Table S4 in Additional file 1. Figure S2 in Additional file 1 shows the number of evaluable cases for each downstream protein. We did not find a significant difference in median phospho-protein expression and relative age of tumor samples (Figure S3a,b,c,d,e in Additional file 1). Median phospho-protein expression was not significantly different among inclusion centers (Figure S4a,b,c,d,e in Additional file 1).

We did not find a significant association between p-p70S6K and any of the known clinico-pathological variables (Table 1). Significant associations were found between p-p70S6K and all other downstream activated proteins (Table 1). The association between other downstream activated proteins and known prognostic variables is shown in Table S5 in Additional file 1. High p-mTOR and positive p-ERK1/2 staining were both associated with a positive PgR status and low tumor grade. In addition, high expression of p-AKT(Ser473) was associated with a positive PgR status.

**Table 1:** Associations between p-p70S6K and clinico-pathological variables as well as other PI3K and/or MAPK pathway proteins

		**p-p70S6K (*n*** **= 438)**		
		**Negative (*n*** **= 188)**	**Positive (*n*** **= 250)**	***P *** **value**
Treatment	No tamoxifen	40 (21)	55 (22)	0.92^a^
	Tamoxifen 1 year	89 (47)	113 (45)	
	Tamoxifen 3 years	59 (31)	82 (33)	
Age	<65	82 (44)	128 (51)	0.12^b^
	≥65	106 (56)	122 (49)	
Lymph node	Negative	92 (49)	141 (56)	0.12^b^
	Positive	96 (51)	109 (44)	
T stage	T1 to T2	167 (89)	221 (88)	1.00^b^
	T3 to T4	21 (11)	29 (12)	
Grade	Grade 1 to 2	123 (65)	153 (61)	0.37^b^
	Grade 3	65 (35)	97 (39)	
Progesterone receptor	Negative	98 (52)	111 (44)	0.10^b^
	Positive	88 (47)	139 (56)	
	Missing	2 (1)	0 (0)	
HER2	Negative	168 (89)	223 (89)	0.17^b^
	Positive	12 (6)	26 (10)	
	Missing	8 (4)	1 (0)	
p-AKT(Ser473)^c^	Low (0 to 1)	109 (71)	53 (24)	<0.0001^b^
	High (2 to 3)	44 (29)	172 (76)	
p-AKT(Thr308)^c^	Negative	143 (81)	87 (36)	<0.0001^b^
	Positive	34 (20)	153 (64)	
p-mTOR^c^	Low (0 to 59%)	156 (87)	176 (73)	0.0003^b^
	High (≥60%)	23 (13)	66 (27)	
p-ERK1/2^c^	Negative	114 (65)	61 (26)	<0.0001^b^
	Positive	61 (35)	177 (74)	

Data presented as *n* (%). ERK, extracellular signal-regulated kinase; HER2, human epidermal growth factor receptor 2; MAPK, mitogen-activated protein kinase; mTOR, mammalian target of rapamycine; PI3K, phosphatidylinositol-3-kinase; p, phosphorylated. ^a^Linear by linear test. ^b^Fisher’s exact test (analysis based on cases without missing values). ^c^Missing data not shown.

### Downstream activated proteins in the PI3K and/or MAPK pathways predict resistance to tamoxifen

**Figure 1:**
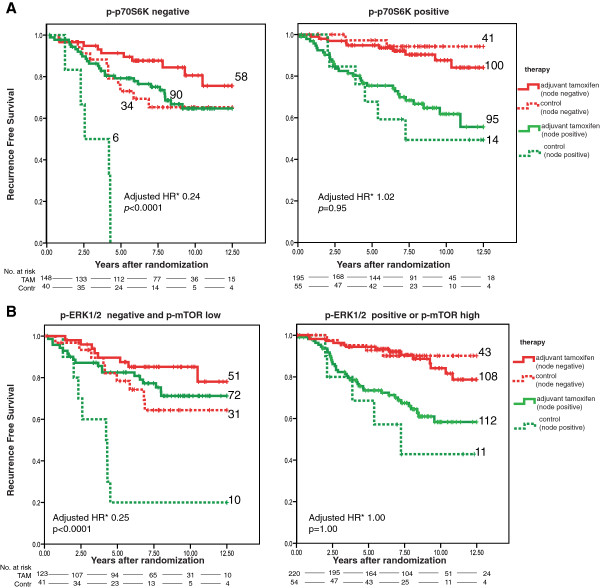
**Kaplan–Meier survival analysis according to tamoxifen treatment and PI3K and/or MAPK pathway marker. (A)** Kaplan–Meier survival analysis according to tamoxifen treatment and p-p70S6K expression: recurrence-free interval according to tamoxifen treatment in patients whose tumors do not express p-p70S6K (left) and patients whose tumors do express p-p70S6K (right). **(B)** Kaplan–Meier survival analysis according to tamoxifen treatment and p-mTOR and p-ERK1/2 expression: recurrence-free interval according to tamoxifen treatment in patients whose tumors do express low p-mTOR and are p-ERK1/2 negative (left) and in patients whose tumors do express p-ERK1/2 and/or express high p-mTOR (right). Contr, control; ERK, extracellular signal-regulated kinase; HR, hazard ratio; MAPK, mitogen-activated protein kinase; mTOR, mammalian target of rapamycine; PI3K, phosphatidylinositol-3-kinase; p, phosphorylated; TAM, tamoxifen.

In the total group of ERα-positive patients (*n* = 563), a total of 132 recurrence-free interval events occurred (Table S6 in Additional file 1). The number of patients in each treatment arm pre and post interim analysis is shown in Figure S5 in Additional file 1. The median follow-up of patients without a recurrence event is 7.8 years. When stratified by nodal status, the hazard ratio (HR) for tamoxifen versus control in this cohort was 0.54 (95% confidence interval (CI) = 0.36 to 0.83, *P* = 0.004). In our primary analysis, using the median expression levels as the cutoff value for a binary factor, we did not find a significant interaction between either p-AKT(Ser473) or p-mTOR and tamoxifen. In addition, the interactions between both p-AKT(Thr308) and p-ERK1/2 and tamoxifen were nonsignificant (adjusted *P* for interaction 0.09 and 0.06 respectively) (Table 2). However, we observed a significant interaction for p-p70S6K with tamoxifen (*P* = 0.004). Patients whose tumor did not express p-p70S6K derived significant benefit from tamoxifen (HR = 0.24, 95% CI = 0.12 to 0.47, *P* < 0.0001), while patients whose tumor did express p-p70S6K had no benefit (multivariate HR = 1.02, 95% CI = 0.48 to 2.21, *P* = 0.95) (Figure 1A; Table S7 in Additional file 1). Performing the same analysis using a 1% cutoff value for ERα positivity added four extra patients and did not substantially change these results (data not shown).

**Table 2:** Adjusted hazard ratios and tests for interaction between PI3K and/or MAPK pathway markers and tamoxifen

	**Variable level**	**Adjusted hazard ratio**^**a **^**for tamoxifen versus control (95% confidence interval)**	**Adjusted *P *** **value for interaction**
p-AKT (Ser473)	Below median (0 to 1)	0.34 (0.17 to 0.69)	0.16
	Above median (2 to 3)	0.68 (0.33 to 1.38)	
p-AKT (Thr308)	Below median (0)	0.42 (0.24 to 0.74)	0.09
	Above median (1 to 3)	1.03 (0.43 to 2.50)	
p-mTOR	Below median (0 to 20%)	0.41 (0.23 to 0.71)	0.25
	Above median (21 to 100%)	0.74 (0.31 to 1.76)	
p-ERK1/2	Below median (0)	0.34 (0.18 to 0.63)	0.06
	Above median (1 to 100%)	0.87 (0.40 to 1.87)	
p-p70S6K	Below median (0)	0.24 (0.13 to 0.47)	0.004
	Above median (1 to 3)	1.02 (0.48 to 2.21)	

ERK, extracellular signal-regulated kinase; MAPK, mitogen-activated protein kinase; mTOR, mammalian target of rapamycine; PI3K, phosphatidylinositol-3-kinase; p, phosphorylated. ^**a**^Recurrence-free interval survival. Models were adjusted for age, T stage, grade, progesterone receptor status and human epidermal growth factor receptor 2 status. Models were stratified for nodal status.

In our exploratory analyses, analyzing the expression of downstream activated proteins as a continuous variable, we observed an interaction with tamoxifen for p-AKT(Thr308) (adjusted *P* = 0.03), p-mTOR (adjusted *P* = 0.03) as well as p-p70S6K (adjusted *P* = 0.006) (Table S8 in Additional file 1). Examining Akaike’s information criteria values indicated that a dichotomization of p-mTOR submembranous staining into <60% and ≥60% provided the best fit to the data (Table S9 and Figure S6 in Additional file 1). For p-AKT(Thr308), p-ERK1/2 and p-p70S6K, a dichotomization in any positive versus negative staining (which corresponded with dichotomization with median expression levels as the cutoff values) provided the best fit to the data (Tables S10,S11 and Figures S7,S8 in Additional file 1). Tables S12,S13,S14,S15 in Additional file 1 present the distribution of known prognostic factors over the treatment arms both for patients who were positive and for patients who were negative for these markers.

Based on preclinical knowledge that p-ERK1/2 and p-mTOR represent activation of, respectively, the MAPK pathway and the PI3K pathway, we explored the interaction with tamoxifen for the combination of these markers. Patients with a tumor that expressed either high p-mTOR or positive p-ERK1/2 protein did not benefit from tamoxifen (adjusted HR = 1.00, 95% CI = 0.48 to 2.08, *P* = 1.00), while patients whose tumor was negative for these two markers did derive significant benefit (adjusted HR = 0.25, 95% CI = 0.13 to 0.48, *P* < 0.0001) (*P* for interaction = 0.004) (Figure 1B; Table S16 in Additional file 1).

### Downstream activated proteins in the PI3K and/or MAPK pathways are associated with good prognosis in the absence of adjuvant systemic treatment

**Figure 2:**
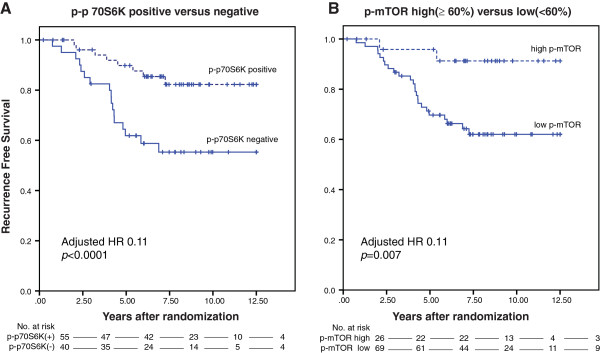
**Kaplan–Meier survival analyses in control patients according to p-p70S6K and p-mTOR.** Kaplan–Meier survival analysis for recurrence-free interval according to **(A)** p-p70S6K and **(B)** p-mTOR. HR, hazard ratio; mTOR, mammalian target of rapamycine; p, phosphorylated.

**Figure 3:**
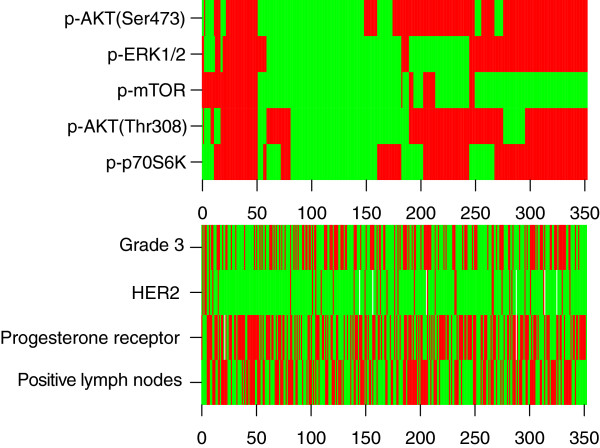
**Unsupervised hierarchical clustering of tumor samples and corresponding expression of PI3K and/or MAPK pathway proteins.** Heat map representing unsupervised hierarchical clustering of tumor samples and corresponding expression of downstream activated proteins in the PI3K and/or MAPK pathways from patients for whom the status of all five proteins were known (*n* = 350). Patients are represented horizontally. Phosphorylated proteins are indicated vertically. Red, high/any expression of phosphorylated protein; green, no/low expression of phosphorylated protein (dichotomization was performed according to Akaike’s information criteria). In addition the presence (red) or absence (green) of different clinico-pathological factors is shown. ERK, extracellular signal-regulated kinase; HER2, human epidermal growth factor receptor 2; MAPK, mitogen-activated protein kinase; mTOR, mammalian target of rapamycine; PI3K, phosphatidylinositol-3-kinase; p, phosphorylated.

When systemically untreated breast cancer patients were dichotomized according to p-AKT(Ser473), p-AKT(Thr308), p-mTOR or p-p70S6K status, the group that was positive for the marker (dichotomized according to Akaike’s information criteria) exhibited a decreased risk for recurrence compared with patients whose tumor had no or low expression of the marker (Figure 2 and Table 3; Tables S17,S18,S19,S20 in Additional file 1). A similar trend was seen for patients whose tumor did express p-ERK1/2 (*P* = 0.14). Hierarchical clustering of the downstream activated proteins in the PI3K and/or MAPK pathway is shown in Figure 3.

**Table 3:** Adjusted hazard ratios for recurrence-free interval according to PI3K and/or MAPK pathway markers in patients who did not receive adjuvant systemic treatment

	**Number of cases in analysis (events)**	**Hazard ratio**^**a**^	**95% confidence interval**	***P *** **value**
**p-AKT(Ser 473), (2 to 3) versus (0 to 1)**	86 (27)	0.30	0.12 to 0.76	0.01
**p-AKT(Thr308), positive versus negative**	100 (26)	0.30	0.11 to 0.80	0.02
**p-mTOR, high (≥60%) versus low (0 to 59%)**	94 (26)	0.11	0.02 to 0.55	0.007
**p-ERK1/2, positive versus negative**	94 (26)	0.52	0.22 to 1.24	0.14
**p-p70S6K, positive versus negative**	94 (25)	0.11	0.04 to 0.32	<0.0001

ERK, extracellular signal-regulated kinase; MAPK, mitogen-activated protein kinase; mTOR, mammalian target of rapamycine; PI3K, phosphatidylinositol-3-kinase; p, phosphorylated. ^a^For each marker, Cox proportional hazard models were performed with co-variables: lymph node status, age, tumor size, grade and progesterone receptor.

## Discussion

In this retrospective analysis of tumor samples from ERα-positive postmenopausal breast cancer patients, we have shown the clinical validity of p-p70S6K, as a marker of PI3K and/or MAPK pathway activation, to predict adjuvant tamoxifen resistance. Patients whose tumor expressed p-p70S6K did not benefit from adjuvant tamoxifen, confirming previous *in vitro* findings [1,2].

We are aware of the discussion that the use of phospho-specific antibodies for IHC may be challenging, since preanalytic variables (such as fixation technique and duration) may critically affect the signal [26]. Although we did not observe an association between phospho-protein expression and either tumor size, inclusion center or relative age of the tumor sample and confirmed the phospho-specificity of the antibodies, we cannot exclude that unknown preanalytic variables might have affected the results of IHC. Nevertheless, since these unknown variables would have affected both tumor samples from control patients as well as tamoxifen-treated patients, the observed predictive value of p-p70S6K markers is probably not biased by these preanalytic variables. Unlike others [29,30], we have used automatic immunostainings to improve robustness and reproducibility of these IHC-based tests. Nevertheless, the visual interpretation of IHC-based tests is still a subjective, time-consuming and variable process, with an inherent intra-observer and inter-observer variability [31,32]. The technical validity of IHC-based tests may therefore be further improved by image analysis applications on digital slides [33]. We deemed a direct comparison of the observed frequencies of breast cancers scoring positive for the downstream-activated proteins analyzed with those observed in other studies [30,34,35] not to be appropriate due to differences in study population, antibodies and staining techniques as well as interpretation of these stainings.

Very few studies have tested the predictive validity of downstream-activated proteins in the PI3K and/or MAPK pathway in the context of a randomized clinical trial. Perez-Tenorio and colleagues observed a decreased benefit from tamoxifen only in those patients with *PIK3CA* mutations who also expressed AKT activation [12], but the test for interaction was not significant. In a subgroup analysis of ERα-positive and PgR-positive patients, Bostner and colleagues recently observed a reduced benefit from tamoxifen in those patients whose tumor expressed high p-mTOR [29]. These results support our data and suggest that downstream activated proteins have a superior clinical validity to predict endocrine resistance compared with the presence or absence of a pathway driver [36]. The strength of our study is that we pre-specified the cutoff value (median expression level) that was used for the primary analysis of the putative marker as a binary factor. In addition, we tested the markers as continuous linear variables and used Aikake’s information criteria to define the optimal cutoff, which coincided with the median for most putative biomarkers. p-p70S6K (as a marker of activation of either the PI3K pathway and/or the MAPK pathway) was the only significant marker according to our conservative definition of significance, although most of the other downstream activated proteins showed a similar trend. In addition, we found a significant interaction for the composed variable of either high p-ERK1/2 and/or p-mTOR expression, representing activated MAPK and PI3K pathways, respectively.

In our study, interestingly, most of the markers of PI3K and/or MAPK activation were associated with a favorable prognosis in the absence of systemic treatment. Although an association with low-grade and positive PgR status was shown, the favorable outcome was independent of these factors in multivariate analysis. The question arises of why these tumors have a favorable prognosis. Although others have studied the association of these markers with prognosis in series of patients treated with endocrine therapy [8,30,37], none of these studies discerned prognosis from prediction. In case a putative biomarker under investigation results in a reduced treatment efficacy, this would cancel out a potential prognostic effect if the biomarker is analyzed in series of patients who are all treated with the drug [10]. A direct comparison between our data and these studies is therefore not possible.

Our study has several limitations. First of all, for retrospective biomarker analysis we could only use the subgroup of 563 ERα-positive patients from whom sufficient tumor material was available. However, this subgroup did not differ from the total study population. Furthermore, the duration of tamoxifen therapy differs from the current duration of at least 5 years. However, we anticipate that the relative effects of the biomarkers analyzed in this study will be similar for shorter and longer duration of endocrine therapy. The patients in our study randomized to adjuvant treatment received tamoxifen only (and no aromatase inhibitors), while currently most ERα-positive postmenopausal breast cancer patients receive an aromatase inhibitor preceding or following tamoxifen treatment. Since the putative predictive biomarkers studied are considered to cause escape from hormone dependency [8], our data are thought to be applicable to current clinical practice as well. Furthermore, the patients in our trial had not received adjuvant chemotherapy and thereby the observed effects of the putative biomarkers were not biased by endocrine-resistant patients who were cured by adjuvant chemotherapy. In current clinical practice, patients who relapse despite adjuvant endocrine therapy and adjuvant chemotherapy are both endocrine therapy resistant and chemotherapy resistant. The adjuvant endocrine therapy resistance may be explained by activation of the PI3K/MAPK pathway and our data suggest that p-p70S6K may identify those patients.

It would be clinically relevant to determine whether p-p70S6K could be used as a companion diagnostic for the addition of non-ERα-targeted drugs (like PI3K/mTOR inhibitors) to endocrine therapy in the adjuvant setting. In metastatic breast cancer patients, who had been randomized between tamoxifen alone or in combination with everolimus, a trend for a better treatment efficacy was observed for patients with a tumor with high p-p70S6K [38]. Randomized clinical trials in the adjuvant setting testing the addition of everolimus to hormonal therapy are currently recruiting patients [39], and these studies are of great importance to test the treatment predictive value of p-p70S6K in this setting.

## Conclusion

With the approval of everolimus for metastatic breast cancer patients with acquired endocrine therapy resistance, the first non-ERα-targeted drug that can overcome endocrine therapy resistance has become clinically available. Whether or not inhibitors of the PI3K/Akt/mTOR pathway may overcome endocrine therapy resistance in the adjuvant setting is currently under investigation. A biomarker of an activated PI3K and/or MAPK pathway with clinical validity to predict endocrine therapy resistance in the adjuvant setting has not been identified [10], but such a biomarker could potentially be used as a companion diagnostic for these non-ERα-targeted drugs. In our series of ERα-positive postmenopausal breast cancer patients, p-p70S6K is significantly associated with intrinsic tamoxifen resistance. Patients whose tumor expresses p-p70S6K, as a marker of downstream PI3K and/or MAPK pathway activation, have a favorable prognosis but do not benefit from adjuvant tamoxifen. It would be of great importance to validate these findings in other randomized series with endocrine therapy and to further explore this marker as a potential companion diagnostic.

## Abbreviations

CI: confidence interval; ERα: estrogen receptor alpha; ERK: extracellular signal-regulated kinase; HER2: human epidermal growth factor receptor 2; HR: hazard ratio; IHC: immunohistochemistry; MAPK: mitogen-activated protein kinase; mTOR: mammalian target of rapamycine; p: phosphorylated; PgR: progesterone receptor; PI3K: phosphatidylinositol-3-kinase; TMA: tissue micro-array.

## Competing interests

The authors declare that they have no competing interests.

## Authors’ contributions

KB, SCL and ADV were responsible for the concept and design of the study. MO, RHTK, JBV, PJvD, JW, and JJM contributed substantially to acquisition of the data. KB, ADV, TMS, EMJJB, SCL, JW and PJvD contributed to the analysis and interpretation of the data. KB, with supervision from SCL, drafted the manuscript. All authors critically revised the manuscript for important intellectual content and approved the final version.

## Supplementary Material

Additional file 1 Is Supplementary Tables S1 to S20 and Supplementary Figures S1 to S8. Click here for file
