# Supplementary material for: Phosphorylated p-70S6K predicts tamoxifen resistance in postmenopausal breast cancer patients randomized between adjuvant tamoxifen versus no systemic treatment
Source: Breast Cancer Res. 2014 Jan 21;16(1):R6. doi: 10.1186/bcr3598 (PMC3979131; doi:10.1186/bcr3598)

Table S1 Distribution of clinic-pathological variables between patients with sufficient tumor material for biomarker analysis and the total group of patients who entered the study

		patients with sufficient tumor material	total study population
		N (%)	*N* (%)
	total	739 (100)	1662 (100)
**age**	<65	378 (51)	869 (52)
	≥ 65	361 (49)	793 (48)
**lymph node**	negative	393 53)	901 (54)
	positive	346 (47)	761 (46)
**T stage**	T 1-2	659 (89)	1482 (89)
	T 3-4	80 (11)	180 (11)
**ER status**[1]	positive	468 (77)	1014 (77)
	negative	142 (23)	311 (23)
**PgR status**[1]	positive	224 (57)	513 (60)
	negative	168 (43)	346 (40)

[1]As defined with ligand binding assay. After revision of recollected tumors, a total of 563 were ERα positive as assessed with immunohistochemistry

Table S2 Antibodies used for immunohistochemical assays

Protein name	Clone	Company	Art. No.	dilution
p-AKT (Ser473)	D9E	Cell signaling	4060	1/50
p-AKT (Thr308)	C31E5E	Cell signaling	2965	1/50
p-ERK1/2 (Thr202/Tyr204)	D13.14.4E	Cell signaling	4370	1/400
p-mTOR (Ser2448)	49F9	Cell signaling	2976	1/300
p-p70S6K(Thr389)	1A5	Cell signaling	9206	1/300

Table S3. Specifications of REMARK recommendations

**Introduction**	
Marker	Activated proteins downstrean in PI3K and/or MAPK pathway (p-AKT(Ser473), p-AKT (Thr308), p-ERK1/2(Thr202/Tyr204), p-mTOR(Ser2448), p-p70S6K(Thr389).
Objectives	To evaluate the predictive capacity of activated proteins downstream in the PI3K and/or MAPK pathway with regard to adjuvant tamoxifen in postmenopausal breast cancer.
Hypothesis	We hypothesize that activated proteins downstream in the PI3K and/or MAPK kinase pathways could potentially be used as a marker that separates patients who are likely to benefit from adjuvant tamoxifen treatment from those who are resistant to this drug.

**Methods (1)**	**Patients**
Characteristics	From 1982 until 1994 a randomized clinical trial was conducted in the Netherlands, studying the benefit of adjuvant tamoxifen (IKA-trial) in postmenopausal breast cancer patients.
Inclusion criteria	In the original study, 1662 breast cancer patients were included who were were post-menopausal, less than 76 years of age and had a $T_{1–4}$, $N_{0–3,}$ M_0 breast tumor. We have traced tissue blocks of participating patients and recollected sufficient tumor material of 739 patients, who did not differ in prognostic factors from the total group (Table S1). After revision of estrogen receptor α (ERα) status as assessed with immunohistochemistry (IHC), a total of 563 ERα positive (≥10%) tumors were used for subsequent analysis.
Exclusion criteria	Mastitis or palpable supra- or infraclavicular lymph nodes
Treatment	Patients were randomized in a 2:1 distribution between 1 year tamoxifen (30 mg per day) versus no adjuvant therapy. After 1 year a second randomization was performed to receive another 2 years of tamoxifen or to stop further treatment. From 1989, based on two interim analyses showing a significant improvement in recurrence free-free survival in lymph node positive patients, these node positive patients were all allocated to the tamoxifen treatment arm (ie skipped the first randomization).

**Methods (2)**	**Specimen characteristics**
Material used	Formalin-fixed paraffin-embedded (FFPE) breast tumor tissue of the primary tumor.
Preservation/storage	Formalin fixation and paraffin embedding. Storage at room temperature.

Methods (3)	Assay methods
Assay	Immunohistochemistry for phospho-proteins in the PI3K and or MAPK pathways was performed using monoclonal antibodies for phospho-AKT(Ser473) (Cell Signaling # 4060)(p-AKT(Ser473)), phospho- AKT(Thr308) (Cell Signaling # 2965)(p-AKT(Thr308)), phospho-mTOR(Ser2448) (Cell Signaling # 2976) (p-mTOR), phospho-p70S6K(Thr 389) (Cell Signalling # 9206)(p-p70S6K) and phospho-p44/42 MAPK(Thr202/Tyr204) (Cell Signaling # 4370) (p-ERK1/2) .
Protocol	For p-AKT (Ser473), antigen retrieval was performed using citrate buffer and slides were incubated overnight with antibody (dilution 1:50). All other stainings were performed using a standardized protocol on the Ventana Benchmark® Ultra system. Staining protocols can be downloaded from our website: http://research.nki.nl/linnlab/index
Control experiments	To ensure phospho-specificity of the antibodies, for each antibody a test TMA containing positive cores was dephosphorylated by λ-phosphatase before staining, resulting in disappearance of the positive staining (Figure S1).
Reproducibility	For each immunohistochemical staining, one of the TMAs was quantified independently in a blinded manner by a second observer to calculate inter-observer variability. The inter-observer variability analyzed using the (weighted) Cohen's kappa coefficient is depicted in Table S4
Quantification	Quantification of immunohistochemical staining was performed as described in the method section for immunohistochemistry.
Blinding	Scoring of the immunohistochemical stainings was done without knowledge regarding both the recurrence-free-interval survival as well as the treatment arm at the time of scoring.

Methods (4)	Study design I
Case selection	A randomized controlled trial. The translational study presented here was performed retrospectively. The median duration of follow-up for patients without a recurrence event was 7.8 years. Patient records were re-evaluated for recurrence until 2000.
Clinical endpoints	The improvement of recurrence free interval (RFI) with tamoxifen versus nil was assessed according to the different levels of the tested drivers and downstream activated proteins as specified below. RFI included local, regional, distant recurrences and breast cancer-specific death, but not contra-lateral breast cancer, as the primary event.
Variables examined or considered	Multivariate Cox models included age (≥ 65 versus < 65), grade (grade 3 versus grade 1-2), tumor size (T3-4 versus T1-T2), HER2 status (positive versus negative) and progesterone status (positive versus negative) as covariates.
Rational for sample size	The sample size of the translational study is based on the amount of available tumor blocks containing invasive, ERα positive tumor cells, that could be recollected and a power calculation based on events in this group assuring that meaningful results could be deduced.

Methods (5)	Statistical analysis
Statistical methods and variable selection procedure	Recurrence free interval was defined as the time from the date of first randomization until the occurrence of a local, regional or distant recurrence or breast cancer specific death. A secondary contra-lateral breast tumor was not considered as an event as explained in the method section and these patients were censored at the date of this occurrence. All calculations were made with Statistical Package for the Social Sciences (SPSS) 15.0 Inc., IL, USA.
Missing data	Cases with a missing value for one of the variables were excluded from the multivariate analysis, with the exception of missing HER2 and PgR data for which a separate level was created
Marker handling in analysis	Our primary analysis was to test whether tamoxifen benefit was dependent on any of the downstream activated proteins in the PI3K and/or /MAPK pathway. Markers were analyzed as binary factor, using the median level as cutoff. Adjusted Cox proportional hazard regression analyses were performed including an interaction variable. Covariates included age (≥ 65 versus < 65), grade (grade 3 versus grade 1-2), tumor size (T3-4 versus T1-T2), HER2 status (positive versus negative), and PgR status (positive versus negative). All survival analyses were stratified for nodal status. We applied a conservative level of significance ($p<0.01$)due to multiple co-primary endpoints.<br><br>Further exploratory analyses examined tamoxifen benefit when the markers were implemented as continuous linear variables. In case an interaction was found, the level of dichotomization that best predicted tamoxifen benefit was tested by comparing the Akaike's Information Criterion (AIC) of the Cox proportional hazards models for all possible cutoffs. In addition, based on knowledge derived from preclinical studies a composed variable of either high p-ERK1/2 or high pmTOR, indicating the activation of either the MAPK or PI3K pathway, was tested for interaction with tamoxifen treatment. Survival curves were constructed using the Kaplan-Meier method.

Results (1)	Data
Flow of patients	See Figure S2 for description of patients excluded for this translational study. See Table S1 for characteristics of total study patients versus the 739 patients with sufficient tumor material included in TMA .
Characteristics	See Table 1 and Table S5.

Results (2)	Analysis and presentation
Relation to standard prognostic variables	See Table 1.
Univariate analysis	See Figures 2, 3, S5-7.
Multivariate analysis:	See Table 2 and Tables S7-10, S17-20. Estimated effects with CIs for marker and all other variables in the model.

Discussion	
Interpretation, limitations and implication	See discussion section

Table S4 Inter-observer variability for antibodies

antibody	scoring system	comparable cores from N patients	weighted Kappa (95% CI)	cutoff used for binary score	Kappa for binary score
**p-AKT(Ser473)**	cytoplasmic intensity	94	0.53 (0.42-0.64)	2-3 versus 0	0.70
**p-AKT(Thr308)**	cytoplasmic intensity	133	0.57 (0.44-0.68)	(1-3) versus 0	0.53
**p-mTOR**	proportion of tumor cells with submembranous staining	101	0.56 (0.44-0.66)	0-59% versus 60% and more	0.60
**p-ERK1/2**	proportion of tumor cells with nuclear staining	69	0.74 (0.60-0.82)	negative versus positive	0.85
**p-p70S6K**	cytoplasmic intensity	97	0.42 (0.25-0.59)	(1-3) versus 0	0.44

Table S5: Association between p-AKT (Ser473), p-AKT (Thr308), p-mTOR, p-ERK1/2 and clinico-pathological variables

		p-AKT(Ser473) (N=394)			p-AKT (Thr308) (N=449)			p-mTOR (N=433)			p-ERK1/2 (N=438)		
		low (0-1) N(%)	high (2-3) N(%)	p-value	negative N(%)	positive N(%)	p-value	low (0-59%) N(%)	high (≥ 60%) N(%)	p-value	negative N (%)	positive N (%)	p-value
**treatment**	**no tamoxifen**	41 (24)	46 (20)	ns	61 (24)	40 (21)	ns	69 (20)	26 (28)	ns	50 (27)	45 (18)	0.04[(1)]
	**tamoxifen 1 yr**	81 (48)	103 (46)		113 (44)	95 (49)		162 (48)	41 (44)		82 (44)	122 (48)	
	**tamoxifen 3 yrs**	46 (27)	77 (34)		82 (32)	58 (30)		109 (32)	26 (28)		53 (29)	86 (34)	
**age**	**<65**	79 (47)	109 (48)	ns	117 (46)	93 (48)	ns	166 (49)	41 (44)	ns	88 (48)	120 (47)	ns
	**≥65**	89 (53)	117 (52)		139 (54)	100 (52)		174 (51)	52 (56)		97 (52)	133 (53)	
**histology**	**ductal**	139 (82)	168 (74)	ns	192 (75)	144 (75)	ns	263 (77)	61 (66)	ns	149 (81)	181 (71)	ns
	**lobular**	13 (8)	16 (7)		25 (10)	14 (7)		28 (8)	12 (13)		16 (9)	20 (8)	
	**others**	16 (10)	42 (18)		39 (15)	35 (18)		49 (14)	20(22)		20 (11)	52 (21)	
**lymph node**	**negative**	84 (50)	125 (55)	ns	139 (54)	103 (53)	ns	174 (51)	54 (58)	ns	97 (52)	136 (54)	ns
	**positive**	84 (50)	101 (45)		117 (46)	90 (47)		166 (49)	39 (42)		88 (48)	117 (46)	
**T stage**	**T1-2**	150 (89)	201 (89)	ns	221 (86)	175 (91)	ns	300 (88)	83 (89)	ns	160 (86)	229 (91)	ns
	**T3-4**	18 (11)	25 (11)		35 (14)	18 (9)		40 (12)	10 (11)		25 (14)	24 (9)	
**grade**	**grade 1-2**	102 (61)	138 (61)	ns	165 (64)	124 (64)	ns	203 (60)	68 (73)	0.02[(2)]	106 (57)	170 (67)	0.04[(2)]
	**grade 3**	66 (39)	88 (39)		91 (36)	69 (36)		137 (40)	25 (27)		79 (43)	83 (33)	
**Progesterone receptor**	**negative**	92 (55)	93 (41)	0.01[(2)]	130 (51)	89 (46)	ns	174 (51)	31 (33)	0.002[(2)]	100 (54)	109 (43)	0.02[(2)]
	**positive**	75 (45)	131 (58)		123 (48)	104 (54)		164 (48)	62 (67)		83 (45)	143 (57)	
	**missing**	1 (1)	2 (1)		3 (1)	0 (0)		2 (1)	0 (0)		2 (1)	1 (0)	
**HER2**	**negative**	149 (89)	201 (89)	ns	229 (90)	172 (89)	ns	301 (89)	87 (94)	ns	168 (91)	223 (88)	ns
	**positive**	15 (9)	20 (9)		20 (8)	17 (9)		32 (9)	4 (4)		12 (6)	26 (10)	
	**missing**	4 (2)	5 (2)		7 (3)	4 (2)		7 (2)	2 (2)		5 (3)	4 (2)	

[1]Linear by linear test
[2]Fisher's exact test; analysis based on cases without missing values

Table S6 Overview of events in 563 ER positive patients

event	total number (%)	first event (%)
Loco (regional) recurrence	25 (4)	21(4)
Distant metastasis	125(22)	110(20)
Secondary contra-lateral breast cancer	23 (4)	21(4)[1]
Breast cancer specific death	87(15)	1(0)

[1]*censored in RFI analysis*

Table S7: Multivariate Cox proportional hazard model of recurrence free interval (RFI) including p-p70S6K and treatment interaction

Variable		Hazard Ratio[1]	95% CI	p-value
**Age**				
< 65	204 (54)	ref		
≥ 65	222 (47)	0.93	0.63-1.39	0.74
**p T-stage**				
T1 or T2	377 (82)	ref		
T3 or T4	49 (19)	1.67	0.99 – 2.81	0.05
**Histologic grade**				
I-II	269 (53)	ref		
III	157 (48)	1.26	0.83-2.08	0.16
**Progesterone receptor**				
negative	205 (45)	ref		
positive	221 (56)	1.35	0.88 – 1.90	0.28
**HER2 status**				
negative	388 (89)	ref		
positive	38 (12)	1.36	0.71– 2.63	0.36
**p-p70S6K**				
negative	179 (52)	ref		
positive	247 (49)	0.22	0.10-0.53	0.001
**Treatment**				
p-p70S6K negative and control	40 (17)	ref		
p-p70S6K negative and tamoxifen	139 (35)	0.24	0.13-0.47	< 0.001
p-p70S6K positive and control	54 (8)	ref		
p-p70S6K positive and tamoxifen	193 (41)	1.02	0.48-2.21	0.95
interaction phospho-S6K X treatment				0.004

[1]stratified for nodal status

Analysis based on 426 cases with 101 events

Table S8: Adjusted p value for the interactions between tamoxifen treatment and downstream activated proteins in the PI3K and/or MAPK pathways analyzed as continuous variable. Covariates included age, T-stage, grade, PgR status and HER2 status. Models are stratified for nodal status

**adjusted interaction tests for markers of PI3K pathway**		
	**variable levels**	**Adjusted p-value for interaction with tamoxifen**
**p-AKT(Ser473)**	**0-3**	0.17
**p-AKT(Thr308)**	**0-3**	0.03
**p-mTOR**	**0-100**	0.03
**p-ERK1/2**	**0-100**	0.14
**p-p70S6K**	**0-3**	**0.006**

Table S9: Multivariate Cox proportional hazard model of recurrence free interval (RFI) including p-mTOR and treatment interaction

**Variable**	**N(events)**	**Hazard Ratio[1]**	**95% CI**	**p-value**
**Age**				
< 65	202 (55)	ref		
≥ 65	219 (48)	0.90	0.60-1.32	0.58
**p T-stage**				
T1 or T2	372 (82)	ref		
T3 or T4	49 (21)	1.72	1.05 – 2.84	0.03
**Histologic grade**				
I-II	264 (54)	ref		
III	157 (49)	1.34	0.88 -2.06	0.18
**Progesterone receptor**				
negative	202 (45)	ref		
positive	219 (58)	1.31	0.86 – 1.99	0.20
**HER2 status**				
negative	385 (91)	ref		
positive	36 (12)	1.45	0.75 -2.78	0.27
**p-mTOR**				
low (0-59%)	330 (86)	ref		
high(60% and more)	91 (17)	0.19	0.05-0.82	0.03
**Treatment**				
low p-mTOR and control	67 (24)	ref		
low p-mTOR and tamoxifen	263 (62)	0.39	0.23-0.64	0.0002
high p-mTOR and control	26 (2)	ref		
high p-mTOR and tamoxifen	65 (15)	2.03	0.46-9.04	0.35
interaction p-mTOR X treatment				0.04

[1]stratified for nodal status

Analysis based on 421 cases with 103 events

Table S10: Multivariate Cox proportional hazard model of recurrence free interval (RFI) including p-AKT(Thr308) and treatment interaction.

		[redacted]		
**Variable**	**N (events)**	**Hazard Ratio[1]**	**95% CI**	**p-value**
**Age**				
< 65	204 (53)	ref		
≥ 65	230 (50)	0.88	0.60-1.31	0.54
**p T-stage**				
T1 or T2	383 (83)	ref		
T3 or T4	51 (20)	1.46	0.88 – 2.43	0.15
**Histologic grade**				
I-II	280 (54)	ref		
III	154 (49)	1.53	1.01-2.32	0.05
**Progesterone receptor**				
negative	215 (46)	ref		
positive	219 (57)	1.26	0.84-1.89	0.27
**HER2 status**				
negative	397 (91)	ref		
positive	37 (12)	1.31	0.68– 2.52	0.42
**p-AKT (Thr308)**				
negative	246 (67)	ref		
positive	188 (36)	0.34	0.13-0.84	0.02
**Treatment**				
p-AKT (Thr308) negative	61 (20)	ref		
p-AKT (Thr308) negative	185 (47)	0.42	0.24-0.74	0.003
p-AKT(Thr308) positive and	39 (6)	ref		
p-AKT(Thr308) positive and	149 (30)	1.03	0.43-2.50	0.94
Interaction p-AKT(Thr308)X treatment				0.09

[1]stratified for nodal status

Analysis based on 434 cases with 103 events

Table S11: Multivariate Cox proportional hazard model of recurrence free interval (RFI) including p-ERK 1/2 and treatment interaction.

Variable	N (events)	Hazard Ratio[1]	95% CI	p-value
**Age**				
< 65	201 (52)	ref		
≥ 65	224 (50)	0.93	0.62-1.38	0.74
**p T-stage**				
T1 or T2	377 (83)	ref		
T3 or T4	48 (19)	1.62	0.96 – 2.72	0.07
**Histologic grade**				
I-II	268 (54)	ref		
III	157 (48)	1.34	0.88-2.05	0.18
**Progesterone receptor**				
negative	206 (44)	ref		
positive	219 (58)	1.32	0.87 – 2.00	0.19
**HER2 status**				
negative	387 (90)	ref		
positive	38 (12)	1.33	0.69- 2.57	0.39
**p-ERK1/2**				
negative	178 (46)	ref		
positive	247 (56)	0.46	0.20-1.08	0.07
**Treatment**				
p-ERK1/2 negative and control	49 (18)	ref		
p-ERK1/2 negative and tamoxifen	129 (28)	0.34	0.18-0.63	0.001
p-ERK1/2 positive and control	45 (8)	ref		
p-ERK1/2 positive and tamoxifen	202 (48)	0.87	0.40-1.86	0.72
Interaction p-ERK1/2 X treatment				0.06

[1]stratified for nodal status

Analysis based on 425 cases with 102 events

Table S12 Patient characteristics by treatment arm and p-mTOR status

		p-mTOR					
		low (0-59%)			high (≥60%)		
		treatment arm			treatment arm		
		control	tamoxifen	p value[1]	control	tamoxifen	p-value[1]
		N (%)	N (%)		N (%)	N (%)	
		69 (100)	271 (100)		26 (100)	67 (100)	
**age**	<65	38 (55)	128 (47)	0.28	12 (46)	29 (43)	0.82
	≥ 65	31 (45)	143 (53)		14 (54)	38 (57)	
**histology**	ductal	54 (78)	209 (77)	0.16	16 (62)	45 (83)	1.00
	lobulars	9 (13)	19 (7)		3 (12)	9 (17)	
	others	6 (9)	43 (16)		7 (26)	13 (19)	
**lymph node**	negative	52 (75)	122 (45)	<0.001	22 (85)	32 (48)	0.001
	positive	17 (25)	149 (55)		4 (15)	35 (52)	
**T stage**	T 1-2	61 (88)	239 (88)	1.00	24 (92)	59 (88)	0.72
	T 3-4	8 (12)	32 (12)		2 (8)	8 (12)	
**grade**	I-II	40 (58)	163 (60)	0.78	18 (69)	50 (75)	0.61
	III	29 (42)	108 (40)		8 (31)	17 (25)	
**progesterone receptor**	negative	37 (54)	137 (51)	0.79	10 (38)	21 (31)	0.63
	positive	32 (46)	132 (49)		16 (62)	46 (69)	
	missing	0 (0)	2 (1)		0 (0)	0 (0)	
**HER2 status**	negative	65 (94)	236 (87)	0.04	25 (96)	62 (93)	1.00
	positive	2 (3)	30 (11)		1 (4)	3 (4)	
	missing	2 (3)	5(2)		0 (0)	2 (3)	

[1]*Fisher's exact test*

Table S13 Patient characteristics by treatment arm and p-p70S6K status

		p-p70S6K					
		negative			positive		
		treatment arm			treatment arm		
		control	tamoxifen	p value[1]	control	tamoxifen	p-value[1]
		N (%)	N (%)		N (%)	N (%)	
		40 (100)	148 (100)		55 (100)	195 (100)	
**age**	<65	25 (63)	57 (39)	0.007	27 (49)	101 (52)	0.76
	≥ 65	15 (38)	91 (61)		28 (51)	94 (48)	
**histology**	ductal	31 (78)	113 (76)	0.40	40 (73)	147 (75)	0.36
	lobulars	6 (15)	14 (9)		5 (9)	11 (6)	
	others	3 (8)	21 (14)		10 (18)	37 (19)	
**lymph node**	negative	34 (85)	58 (39)	<0.001	41 (75)	100 (51)	0.002
	positive	6 (15)	90 (61)		14 (25)	95 (49)	
**T stage**	T 1-2	37 (93)	130 (88)	0.57	50 (91)	171 (88)	0.54
	T 3-4	3 (8)	18 (12)		5 (9)	24 (12)	
**grade**	I-II	23 (58)	100 (68)	0.26	35 (64)	118 (61)	0.76
	III	17 (43)	48 (32)		20 (36)	77 (39)	
**progesterone receptor**	negative	24 (60)	74 (50)	0.37	22 (40)	89 (46)	0.54
	positive	16 (40)	72 (49)		33 (60)	106 (54)	
	missing	0 (0)	2 (1)		0 (0)	0 (0)	
**HER2 status**	negative	39 (98)	129 (87)	0.31	51 (93)	172 (88)	0.22
	positive	1 (3)	11 (7)		3 (5)	23 (12)	
	missing	0 (0)	8(5)		1 (2)	0 (0)	

[1]*Fisher's exact test*

Table S14 Patient characteristics by treatment arm and p-ERK1/2 status

		p-ERK1/2					
		negative			positive		
		treatment arm			treatment arm		
		**control**	**tamoxifen**	**p value**[1]	**control**	**tamoxifen**	**p-value**[1]
		N (%)	N (%)		N (%)	N (%)	
		50 (100)	135 (100)		45 (100)	208 (100)	
**age**	<65	27 (54)	61 (45)	0.32	24 (53)	96 (46)	0.41
	≥ 65	23 (46)	74 (55)		21 (47)	112 (54)	
**histology**	ductal	41 (82)	108 (80)	0.40	29 (64)	152 (73)	0.13
	lobulars	6 (12)	10 (7)		6 (13)	14 (7)	
	others	3 (6)	17 (13)		10(22)	42(20)	
**lymph node**	negative	39 (78)	58 (43)	<0.001	35 (78)	101 (49)	<0.001
	positive	11 (22)	77 (57)		10 (22)	107 (51)	
**T stage**	T 1-2	43 (86)	117 (87)	1.00	42 (93)	187 (90)	0.59
	T 3-4	7 (14)	18 (13)		3 (7)	21 (10)	
**grade**	I-II	25 (50)	81 (60)	0.24	33 (73)	137 (66)	0.38
	III	25 (50)	54 (40)		12 (27)	71 (34)	
**progesterone receptor**	negative	29 (58)	71 (53)	0.62	18 (40)	91 (44)	0.74
	positive	21 (42)	62 (46)		27 (60)	116 (56)	
	missing	0 (0)	2 (1)		0 (0)	1 (0)	
**HER2 status**	negative	48 (96)	120 (89)	0.18	43 (96)	180 (87)	0.18
	positive	1 (2)	11 (8)		2 (4)	24 (12)	
	missing	1(2)	4 (3)		0 (0)	4(1)	

[1]*Fisher's exact test*

Table S15 Patient characteristics by treatment arm and p-AKT (Thr308) status

		p-AKT (Thr308)					
		**negative**			**positive**		
		**treatment arm**			**treatment arm**		
		**control**	**tamoxifen**	**p value[1]**	**control**	**tamoxifen**	**p-value[1]**
		N (%)	N (%)		N (%)	N (%)	
		61 (100)	195 (100)		40 (100)	153 (100)	
**age**	<65	31 (51)	86 (44)	0.38	21 (53)	72 (47)	0.60
	≥ 65	30 (49)	109 (56)		19 (48)	81 (53)	
**histology**	ductal	46 (75)	146 (75)	1.00	28 (70)	116 (76)	0.17
	lobulars	6 (10)	19 (10)		5 (13)	9 (6)	
	others	9 (15)	30 (15)		7 (18)	28 (18)	
**lymph node**	negative	51 (84)	88 (45)	<0.001	28 (70)	75 (49)	0.02
	positive	10 (16)	107 (55)		12 (30)	78 (51)	
**T stage**	T 1-2	52 (85)	169 (87)	0.83	38 (95)	137 (90)	0.37
	T 3-4	9 (15)	26 (13)		2 (5)	16 (10)	
**grade**	I-II	34 (56)	131 (67)	0.13	30 (75)	94 (61)	0.14
	III	27 (44)	64 (33)		10 (25)	59 (39)	
**progesterone receptor**	negative	34 (56)	96 (49)	0.47	18 (45)	71 (47)	1.00
	positive	27 (44)	96 (49)		22 (55)	82 (54)	
	missing	0(0)	3 (2)		0 (0)	0 (0)	
**HER2 status**	negative	59 (97)	170 (87)	0.17	38 (95)	134 (88)	0.20
	positive	2 (3)	18 (9)		1 (3)	16 (10)	
	missing	0 (0)	7 (4)		1 (3)	3 (2)	

[1]*Fisher's exact test*

Table S16. Multivariate Cox proportional hazard model of recurrence free interval (RFI) including p-mTOR or p-ERK1/2 and treatment interaction.

Variable	N(events)	Hazard Ratio[1]	95% CI	p-value
**Age**				
< 65	201 (52)	ref		
≥ 65	224 (50)	0.93	0.63-1.39	0.74
**p T-stage**				
T1 or T2	377 (83)	ref		
T3 or T4	48 (19)	1.70	1.01 – 2.85	0.05
**Histologic grade**				
I-II	268 (54)	ref		
III	157 (48)	1.32	0.87 -2.03	0.20
**Progesterone receptor**				
negative	206 (44)	ref		
positive	219 (58)	1.34	0.88 – 2.03	0.17
**HER2 status**				
negative	387 (90)	ref		
positive	38 (12)	1.32	0.69 -2.56	0.40
**p-mTOR and/or p-ERK1/2**				
p-mTOR low (0-59%) and pERK1/2 negative	157 (41)	ref		
p-mTOR high(60% and more) or pERK1/2 positive	268 (61)	0.34	0.15-0.77	0.01
**Treatment**				
(low p-mTOR and pERK/2 negative) and control	40 (17)	ref		
(low p-mTOR and pERK/2 negative) and tamoxifen	117 (24)	0.25	0.13-0.48	<0.0001
(high p-mTOR or pERK/2 positive) and control	54 (9)	ref		
(high p-mTOR or pERK/2 positive) and tamoxifen	214 (52)	1.00	0.48-2.08	1.00
Interaction p-mTOR X treatment				0.004

[1]stratified for nodal status

Analysis based on 425 cases with 102 events

Table S17. Multivariate Cox proportional hazard model of recurrence free interval (RFI) including p-p70S6K in patients who did not receive tamoxifen

Variable	N(events)	Hazard Ratio	95% CI	p-value
**Age**				
< 65	52 (16)	ref		
≥ 65	42 (9)	0.75	0.32-1.77	0.51
**p T-stage**				
T1 or T2	87 (20)	ref		
T3 or T4	7 (5)	6.45	1.87 – 22.17	0.003
**Histologic grade**				
I-II	57 (14)	ref		
III	37 (11)	0.76	0.33-1.76	0.53
**Progesterone receptor**				
negative	45 (11)	ref		
positive	49 (14)	1.31	0.55-3.18	0.54
**Lymph node status**				
negative	75 (13)	ref		
positive	19 (12)	7.47	2.80– 19.90	<0.001
**p-p70S6K**				
negative	40 (17)	ref		
positive	54 (8)	0.11	0.04-0.32	<0.001
Analysis based on 94 cases with 25 events				

Table S18. Multivariate Cox proportional hazard model of recurrence free interval (RFI) including p-mTOR in patients who did not receive tamoxifen

Variable	N(events)	Hazard Ratio	95% CI	p-value
**Age**				
< 65	50 (16)	ref		
≥ 65	44 (10)	0.52	0.21-1.27	0.15
**p T-stage**				
T1 or T2	85 (20)	ref		
T3 or T4	9 (6)	5.79	1.88-17.82	0.002
**Histologic grade**				
I-II	57 (14)	ref		
III	37 (12)	0.85	0.35-2.09	0.73
**Progesterone receptor**				
negative	46 (11)	ref		
positive	48 (15)	1.91	0.75-4.85	0.17
**Lymph node status**				
negative	74 (13)	ref		
positive	20 (13)	4.47	1.90– 10.52	0.001
**p-mTOR**				
Low (0-59%)	68 (24)	ref		
High (≥ 60%)	26 (2)	0.11	0.02-0.55	0.007
Analysis based on 94 cases with 26 events				

Table S19. Multivariate Cox proportional hazard model of recurrence free interval (RFI) including p-AKT(Thr308) in patients who did not receive tamoxifen

**Variable**	**N(events)**	**Hazard Ratio**	**95% CI**	**p-value**
**Age**				
< 65	52 (16)	ref		
≥ 65	48 (10)	0.52	0.29-1.17	0.12
**p T-stage**				
T1 or T2	90 (20)	ref		
T3 or T4	10 (6)	1.62	0.54 – 4.88	0.40
**Histologic grade**				
I-II	63 (14)	ref		
III	37 (12)	1.17	0.49-2.76	0.73
**Progesterone receptor**				
negative	51 (11)	ref		
positive	49 (15)	1.18	0.50-2.80	0.71
**Lymph node status**				
negative	79 (13)	ref		
positive	21 (13)	5.76	2.25– 14.75	0.0002
**p-AKT (Thr308)**				
negative	61 (20)	ref		
positive	39 (6)	0.30	0.12-0.80	0.02

Analysis based on 100 cases with 26 events

Table S20. Multivariate Cox proportional hazard model of recurrence free interval (RFI) including p-AKT(Ser473) in patients who did not receive tamoxifen

		[redacted]		
**Variable**	**N(events)**	**Hazard Ratio**	**95% CI**	**p-value**
**Age**				
< 65	44 (14)	ref		
≥ 65	43 (10)	0.55	0.23-1.32	0.19
**p T-stage**				
T1 or T2	79 (20)	ref		
T3 or T4	8 (4)	8.98	2.17-37.10	0.002
**Histologic grade**				
I-II	44 (14)	ref		
III	43 (10)	0.45	0.47-1.12	0.11
**Progesterone receptor**				
negative	44 (11)	ref		
positive	43 (13)	1.51	0.61-3.71	0.37
**Lymph node status**				
negative	67 (21)	ref		
positive	20 (12)	7.23	2.88-18.12	<0.001
**p-AKT (Ser473)**				
negative	41 (14)	ref		
positive	46 (10)	0.30	0.12-0.76	0.01

Analysis based on 87 cases with 24 events

Figure S1 phospho-specificity of the antibodies

For each antibody a test TMA containing positive cores was dephosphorylated by λ-phosphatase before staining, resulting in no detection of the phospho-protein.

A. p-AKT(Ser473) without λ-phosphatase (left panel) and after λ-phosphatase (right panel)
B. p-AKT(Thr 308) without λ-phosphatase (left panel) and after λ-phosphatase (right panel)
C. p-mTOR(Ser2448) without λ-phosphatase (left panel) and after λ-phosphatase (right panel)
D. p-ERK1/2 (Thr202/Tyr204) without λ-phosphatase (left panel) and after λ-phosphatase (right panel)
E. p-p70S6K(Thr389) without λ-phosphatase (left panel) and after λ-phosphatase (right panel)

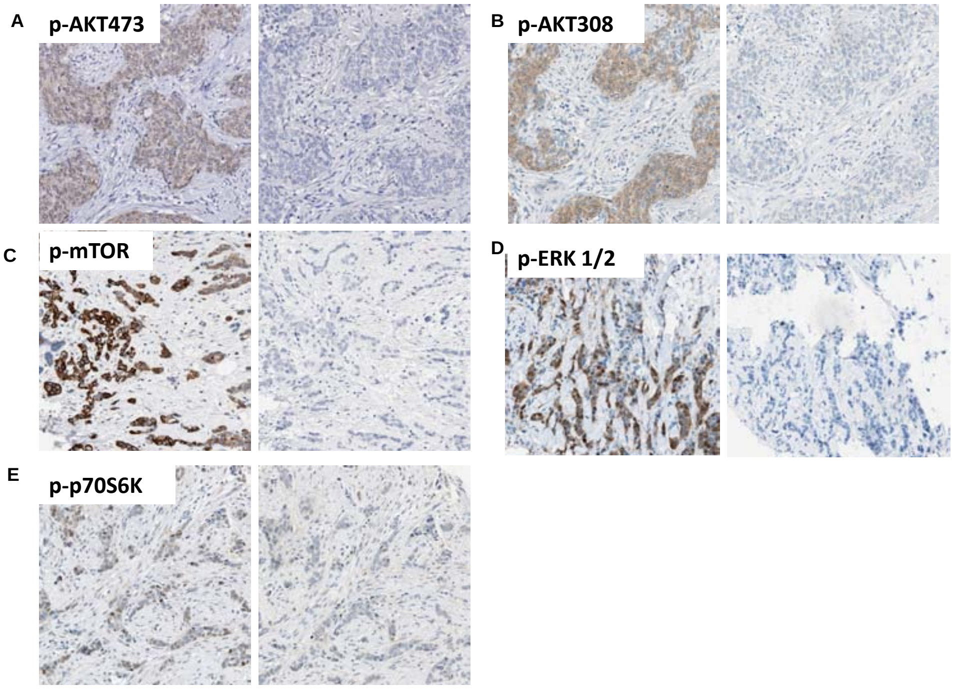

Figure S2 Data flow

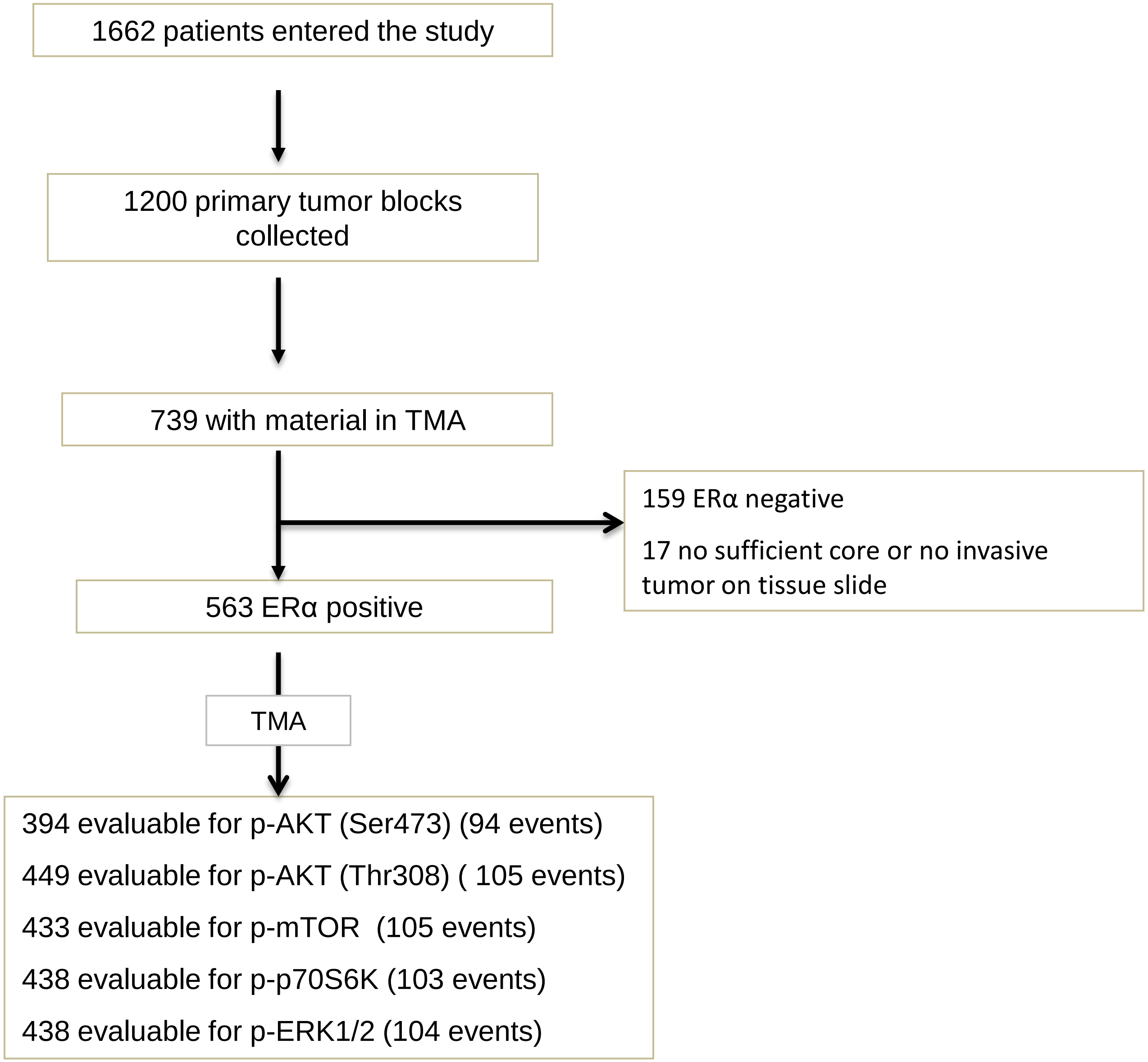

Figure S3a: Expression of p-AKT (ser473) according to relative age of tumor samples (divided in quartiles)

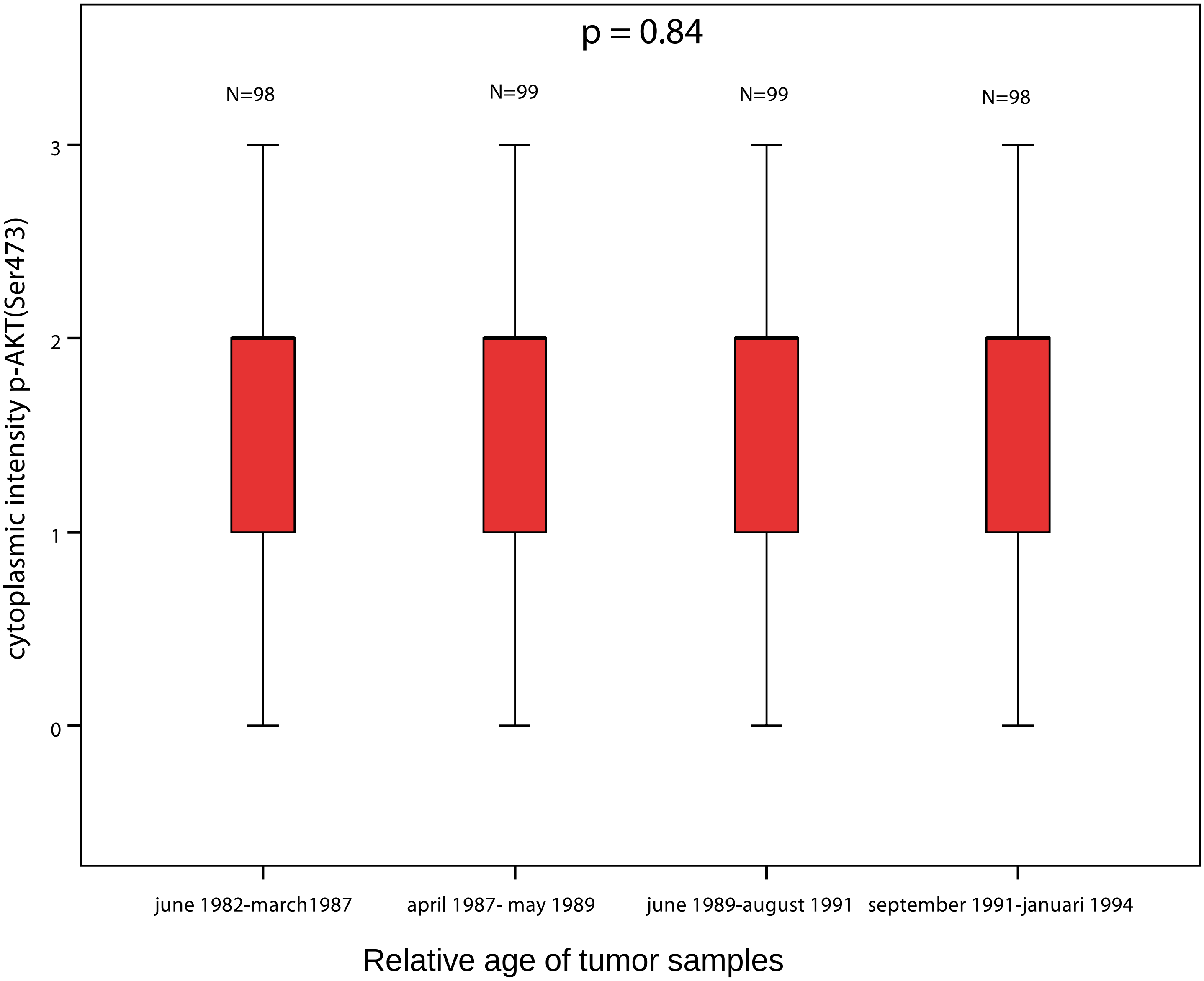

Figure S3b: Expression of p-AKT (Thr308) according to relative age of tumor samples (divided in quartiles)

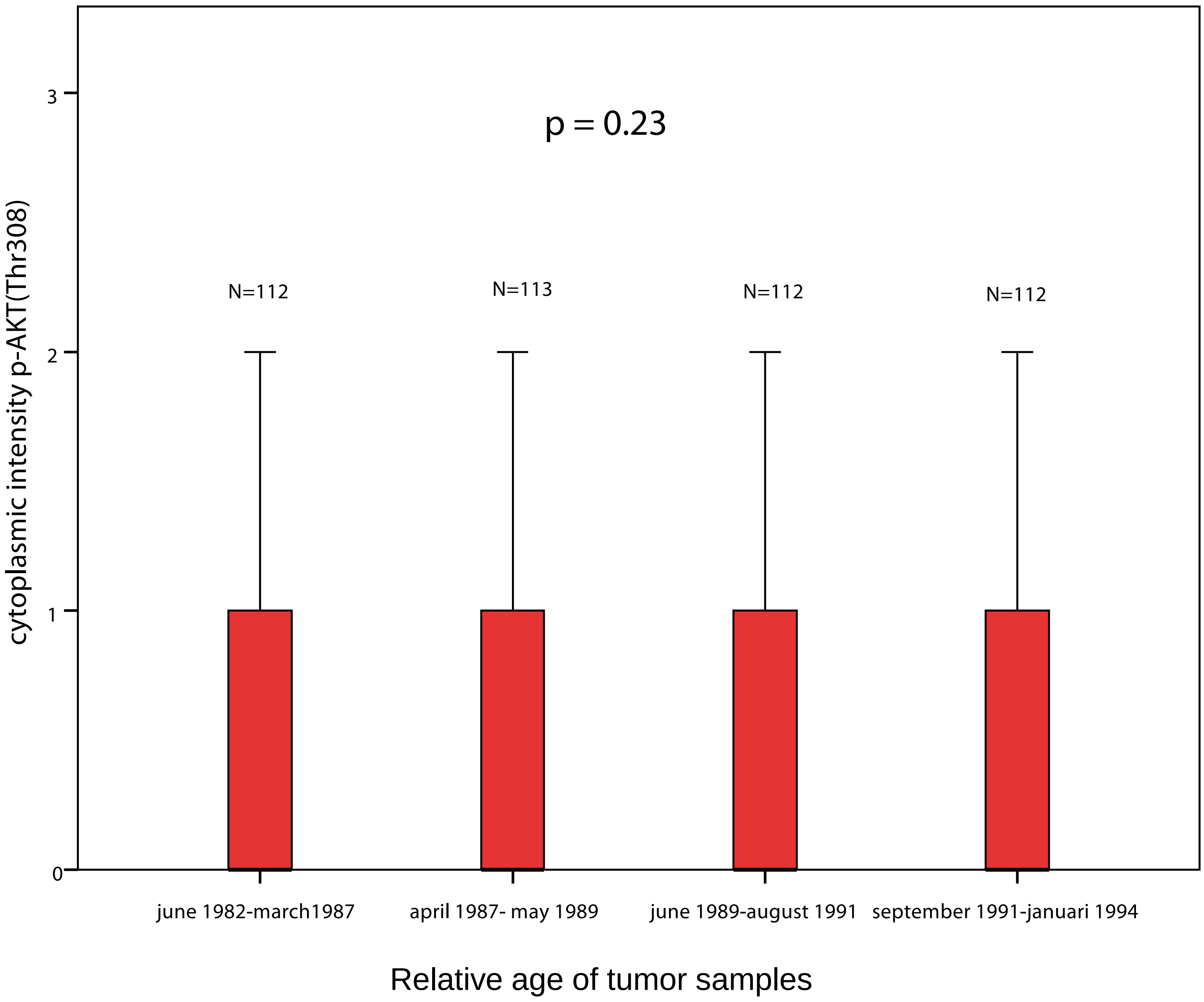

Figure S3c Expression of p-mTOR according to relative age of tumor samples (divided in quartiles)

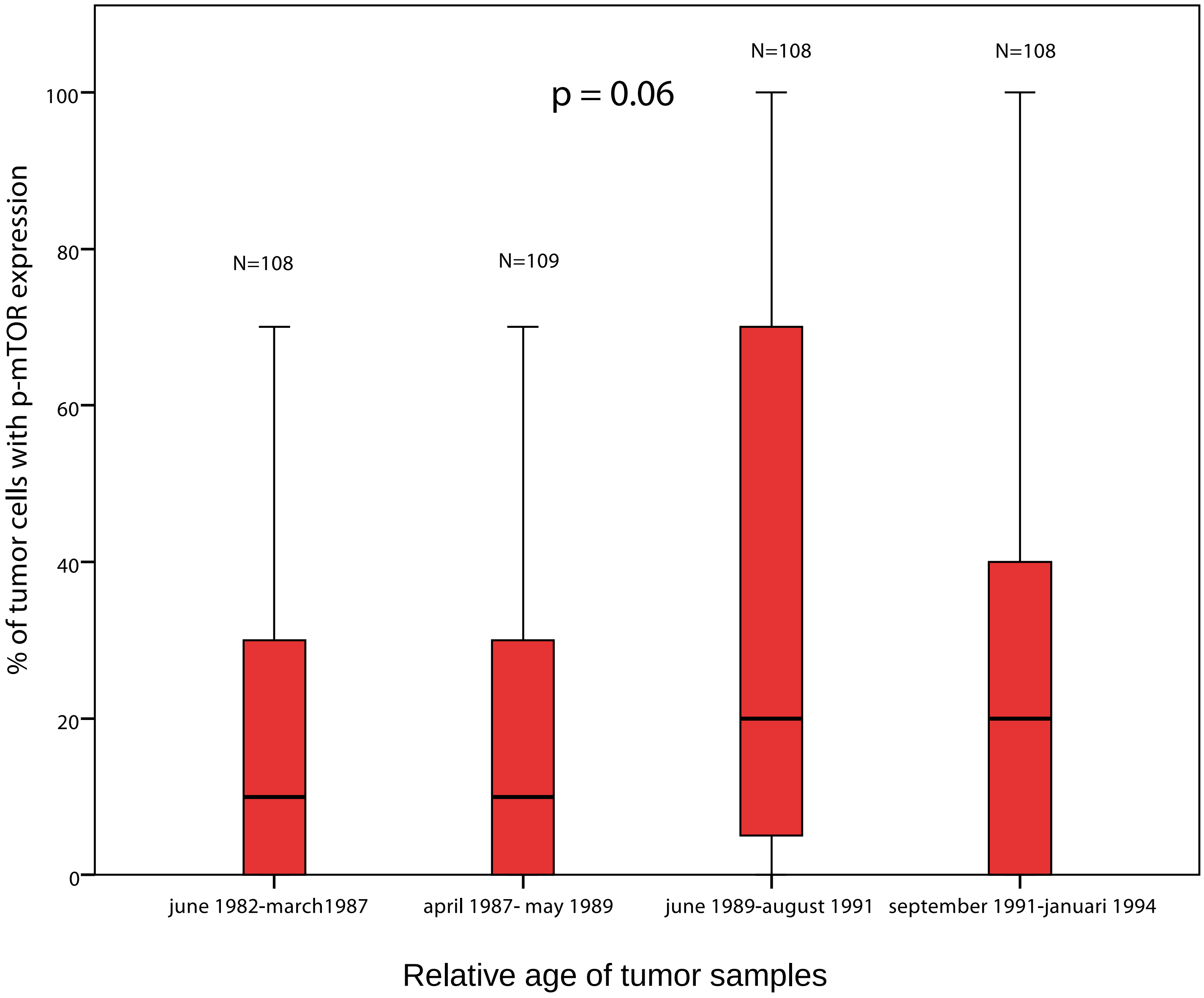

Figure S3d Expression of p-ERK1/2 according to relative age of tumor samples (divided in quartiles)

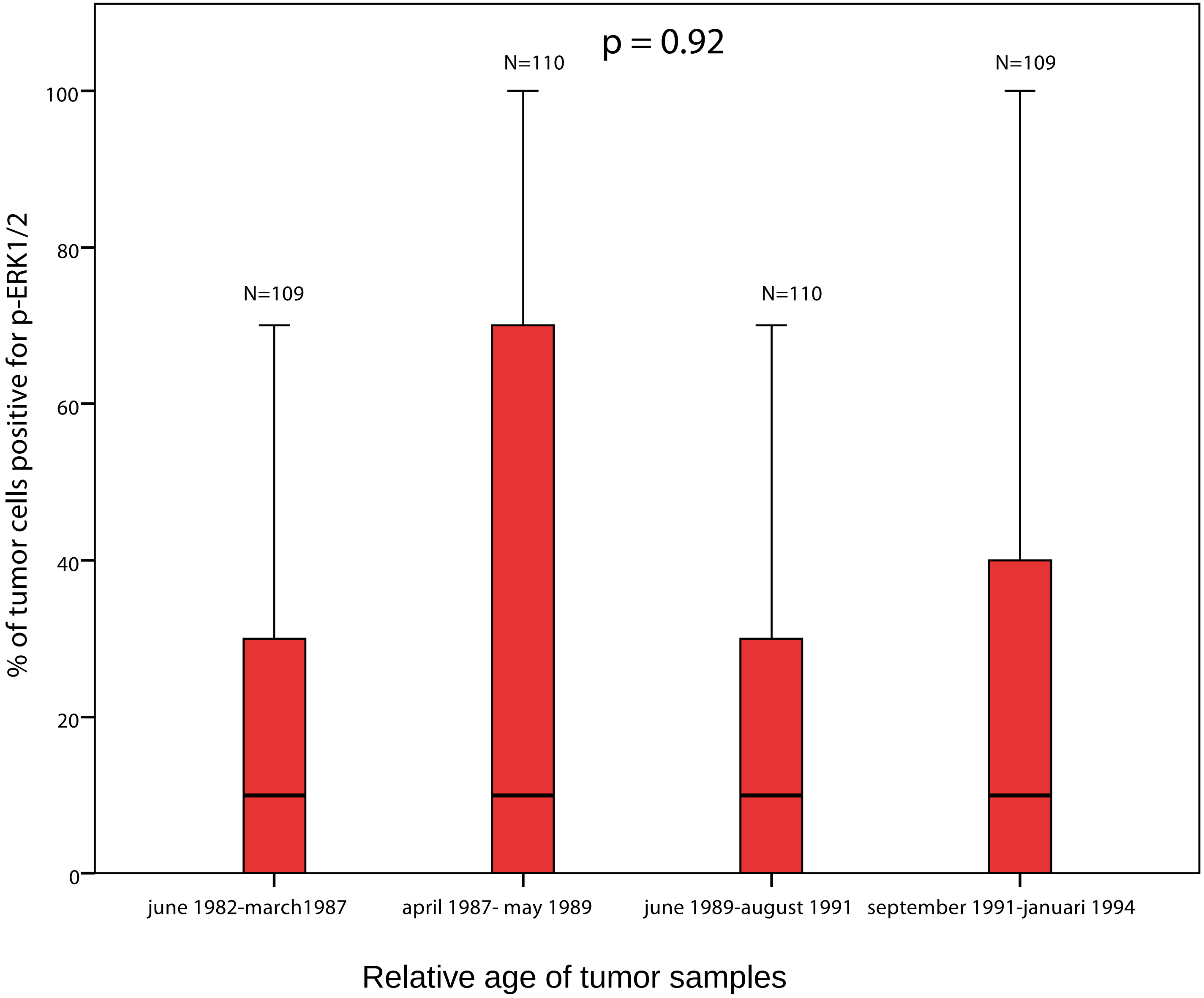

Figure S3e Expression of p-p70S6K according to relative age of tumor samples (divided in quartiles)

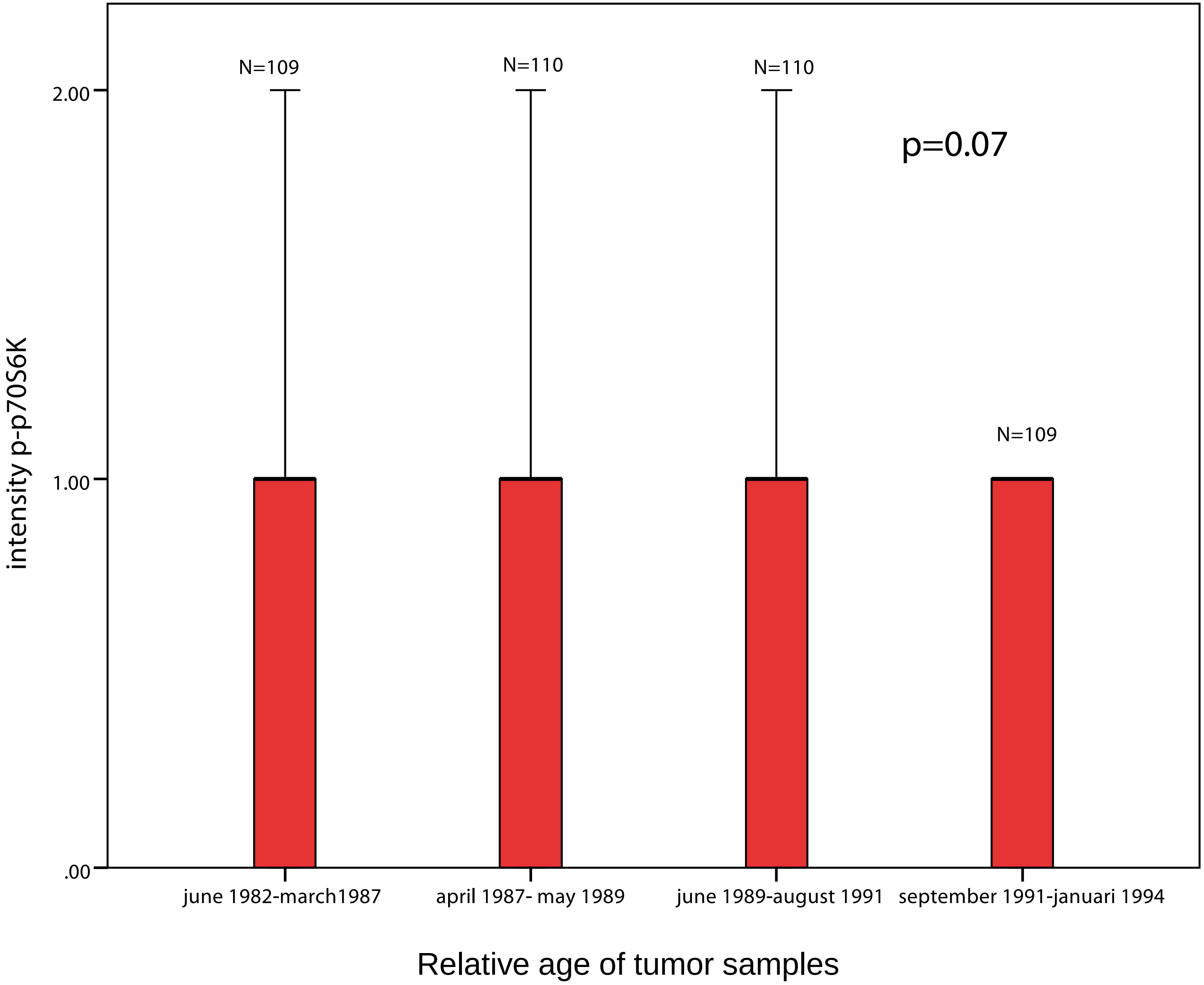

Figure S4a Expression of p-AKT (Ser473) according to institute*

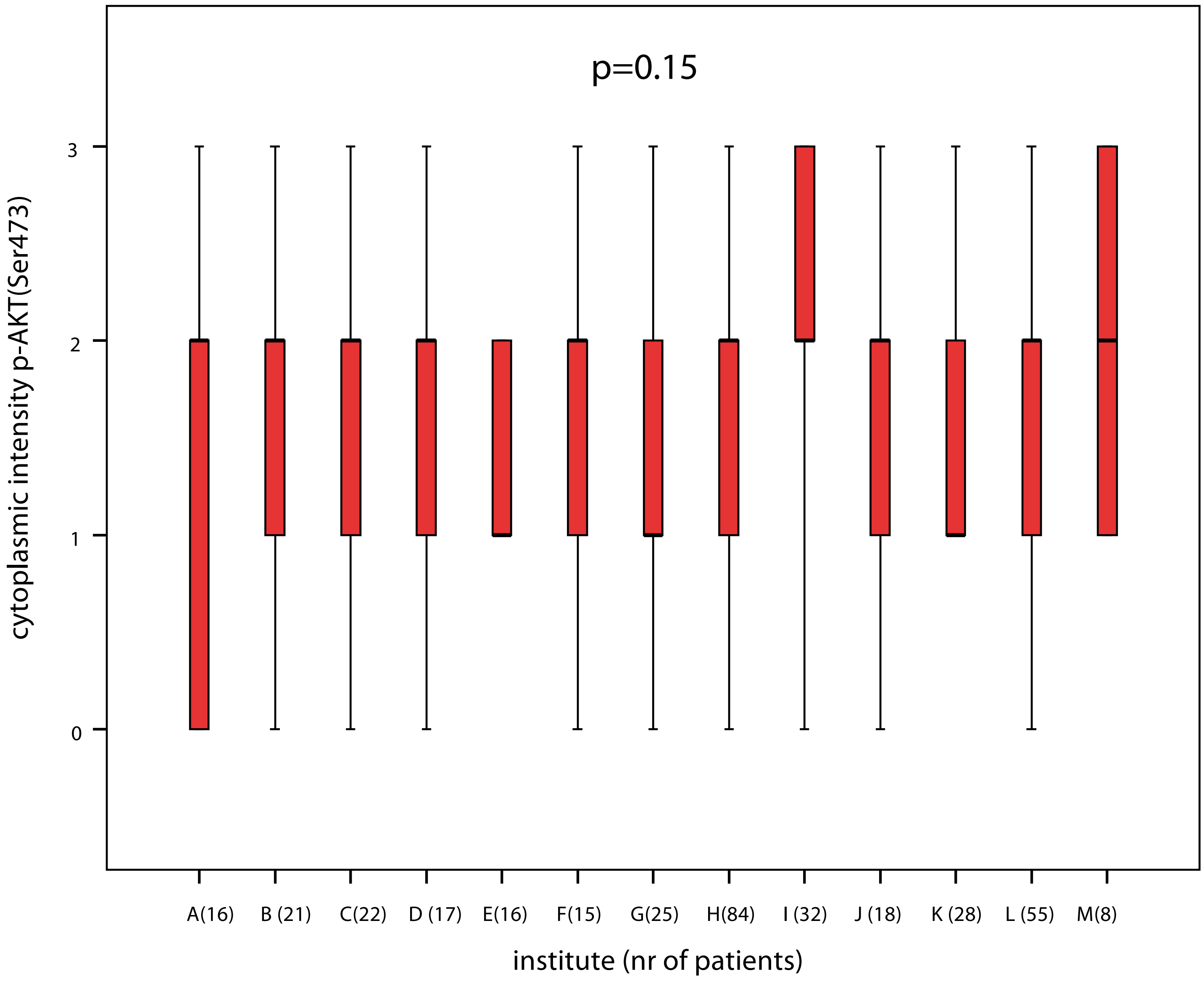

*institutes including ≥ 10 patients with sufficient data for analysis of at least one phospho-protein are shown

Figure S4b Expression of p-AKT (Thr308) according to institute*

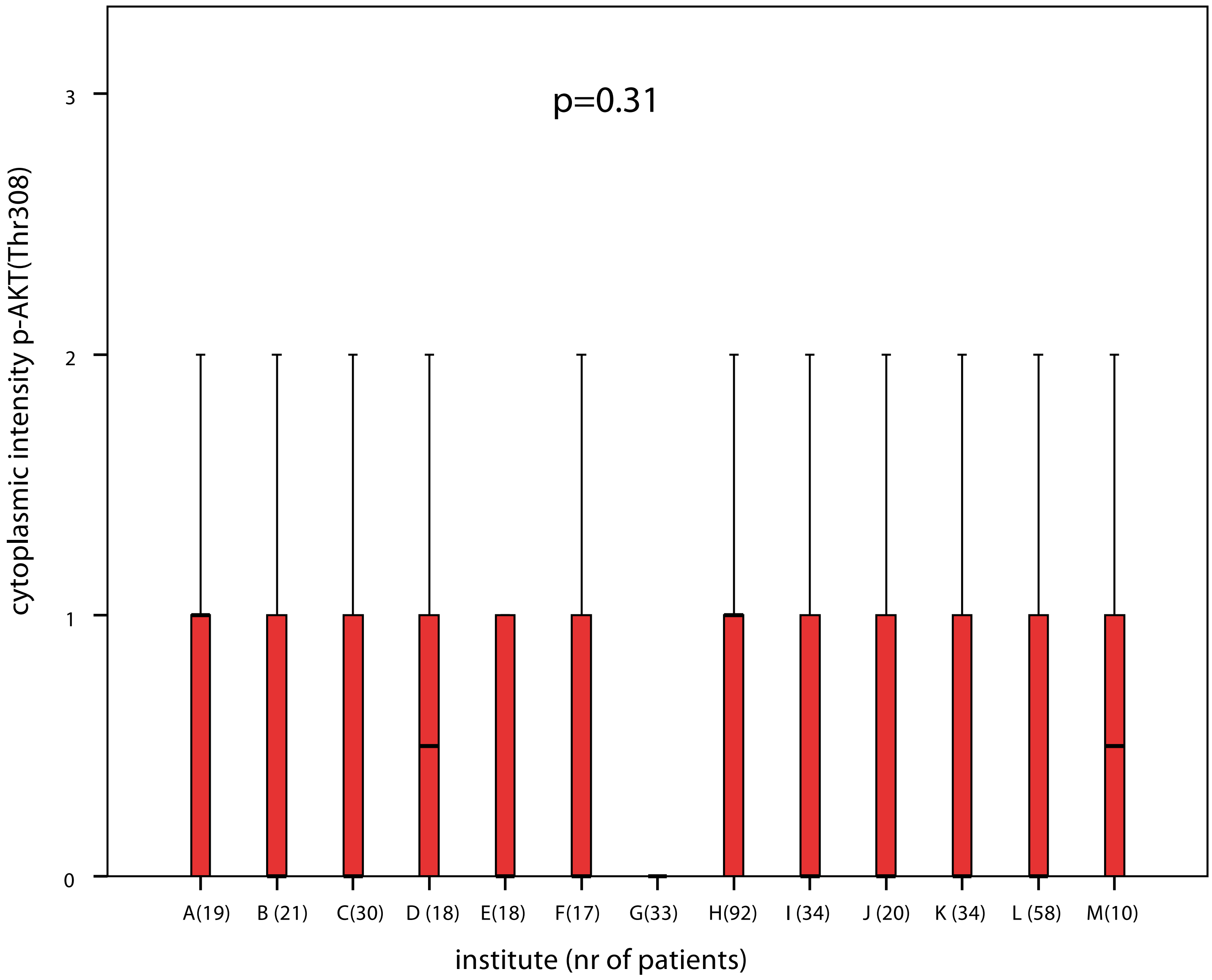

*institutes including ≥ 10 patients with sufficient data for analysis of at least one phospho-protein are shown

Figure S4c Expression of p-mTOR according to institute*

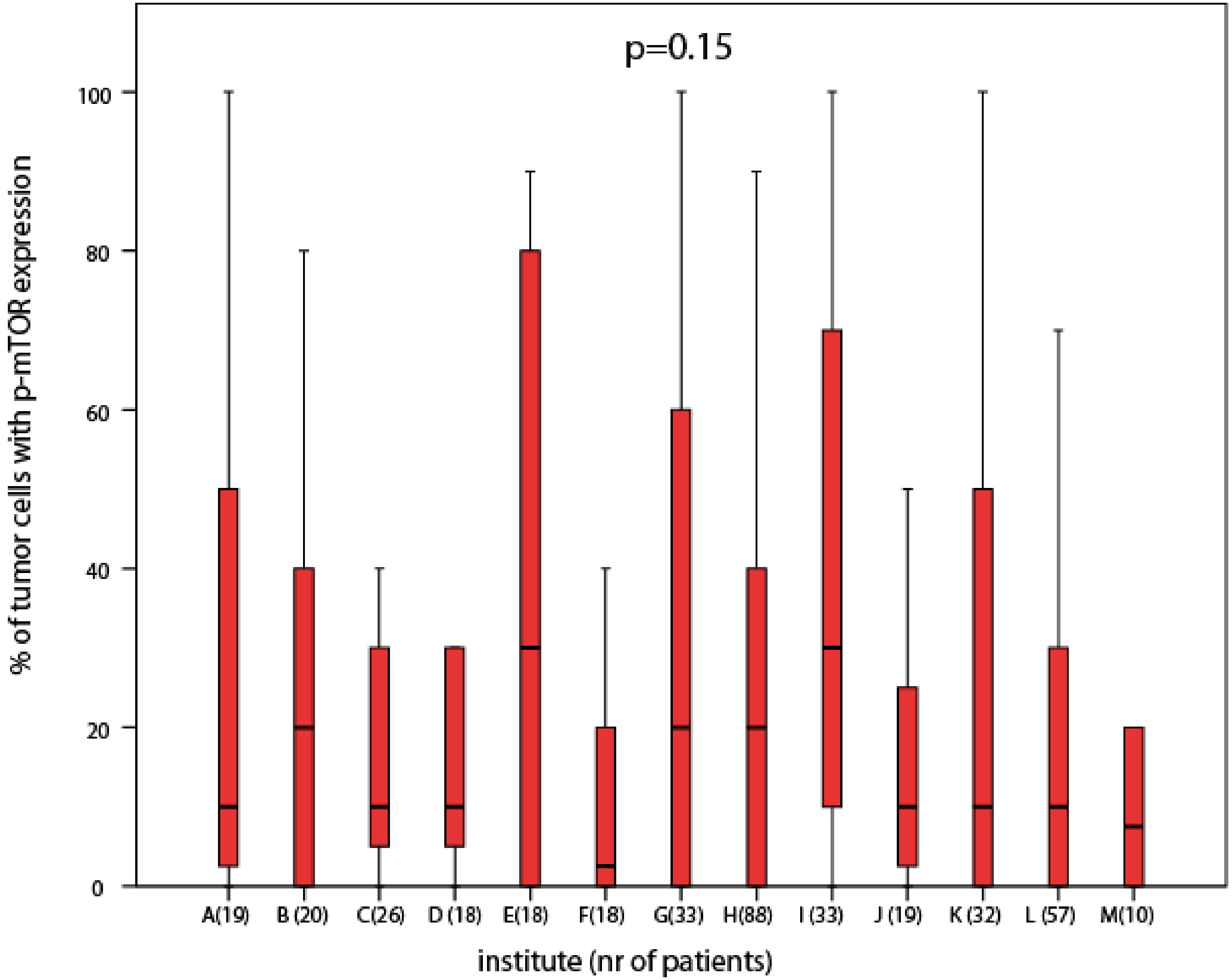

*institutes including ≥ 10 patients with sufficient data for analysis of at least one phospho-protein are shown

Figure S4d Expression of p-ERK1/2 according to institute*

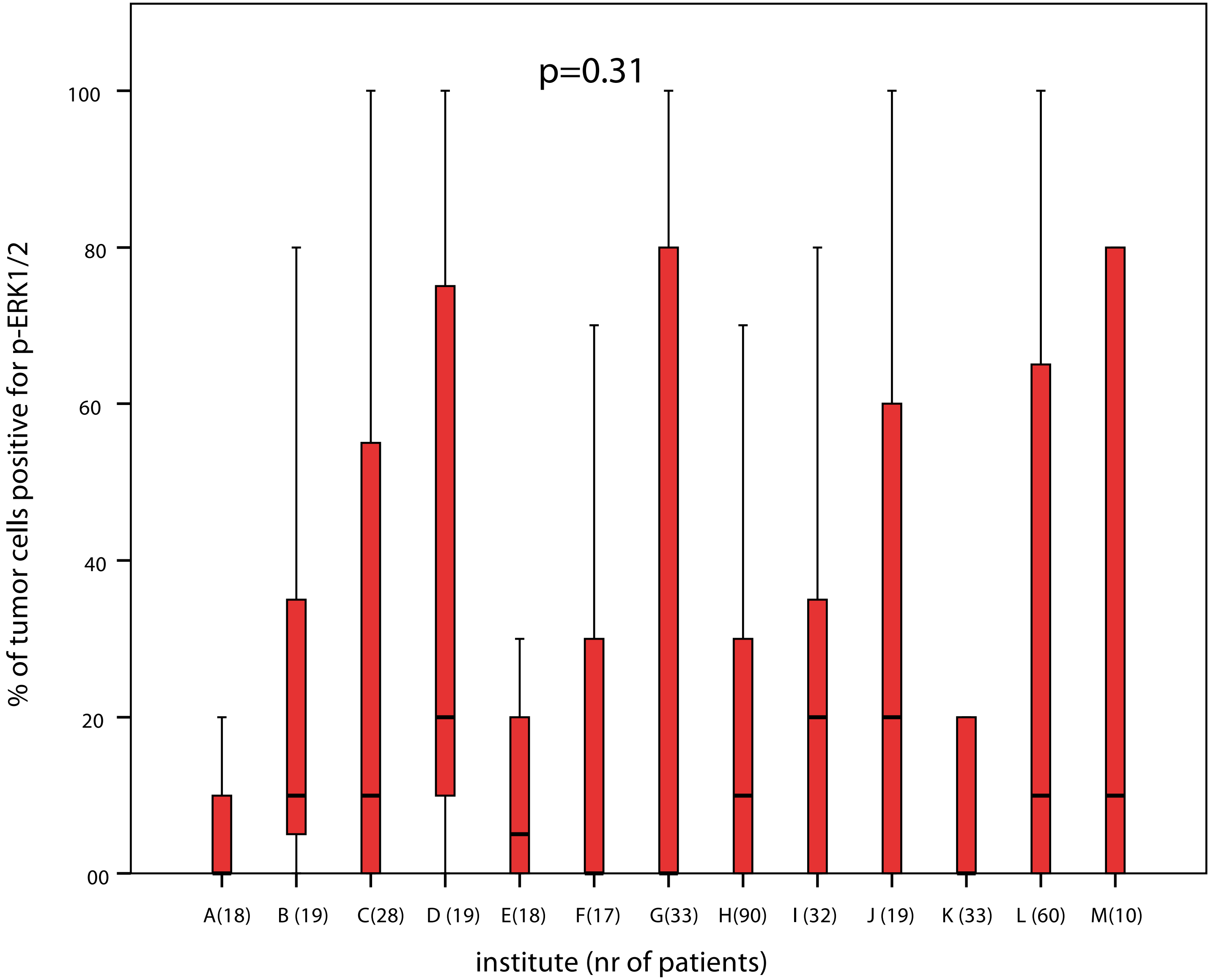

*institutes including ≥ 10 patients with sufficient data for analysis of at least one phospho-protein are shown

Figure S4e Expression of p-p70S6K according to institute*

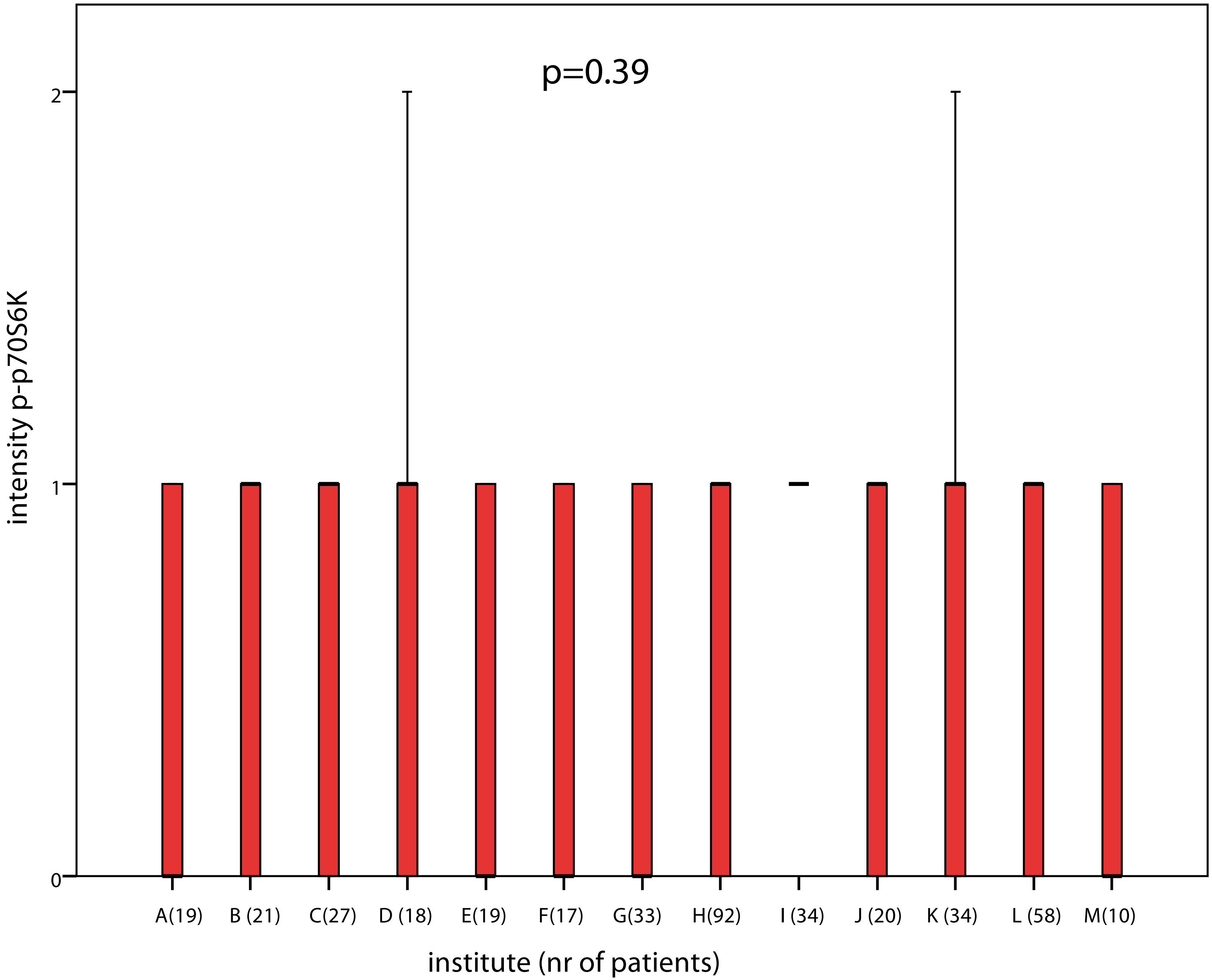

*institutes including ≥ 10 patients with sufficient data for analysis of at least one phospho-protein are shown

Figure S4F Expression of PgR according to institute*

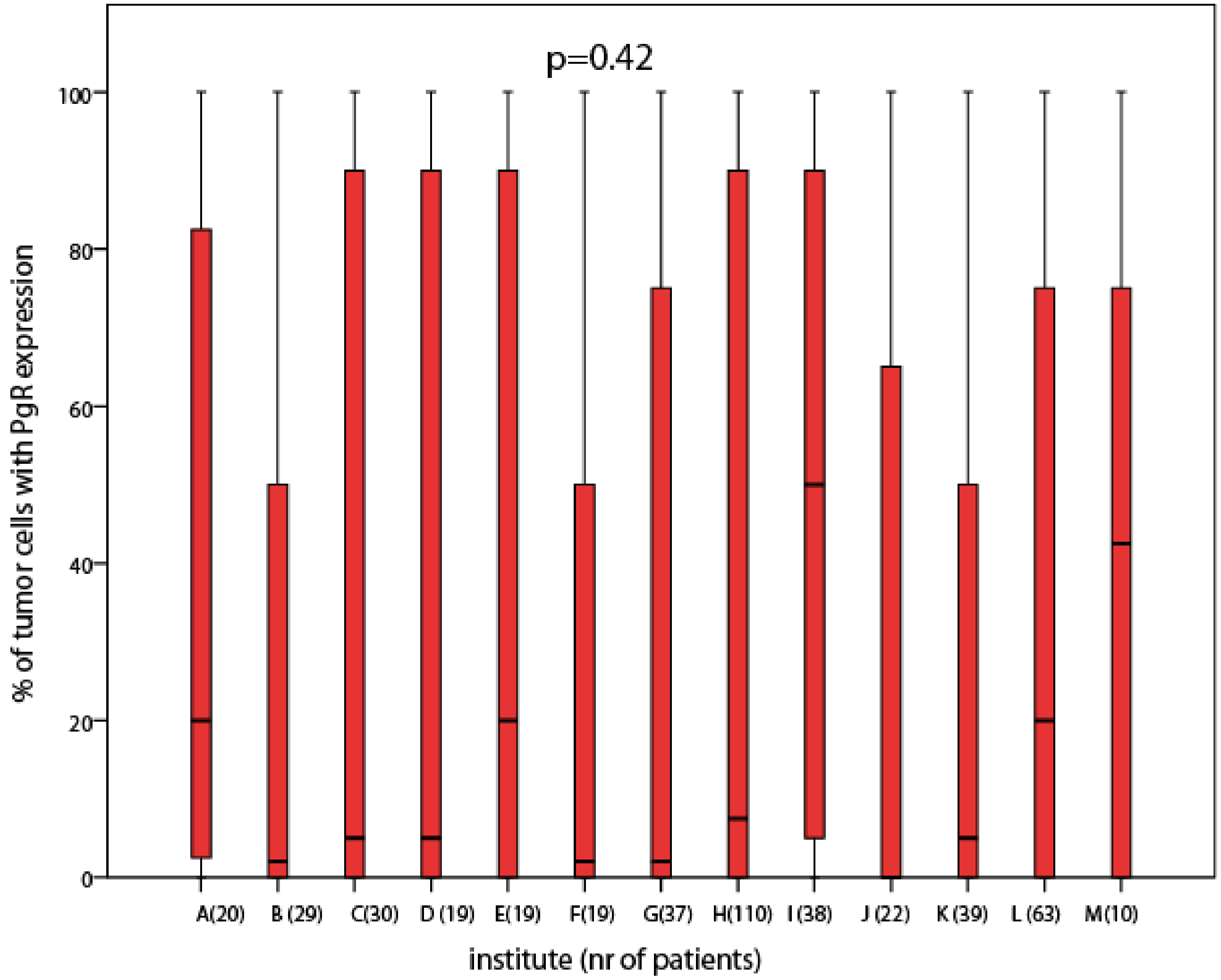

*institutes including ≥ 10 patients with sufficient data for analysis of at least one phospho-protein are shown

Figure S5A. Numbers per treatment group pre-interim analysis

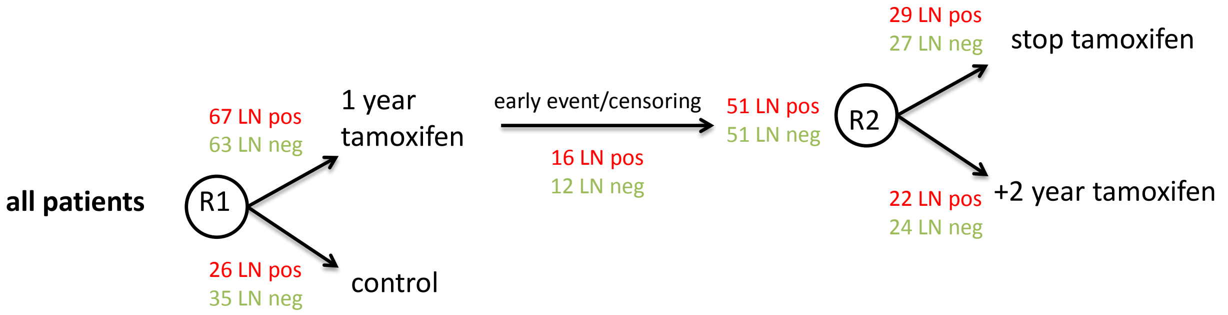

Figure S5B. Numbers per treatment group post-interim analysis

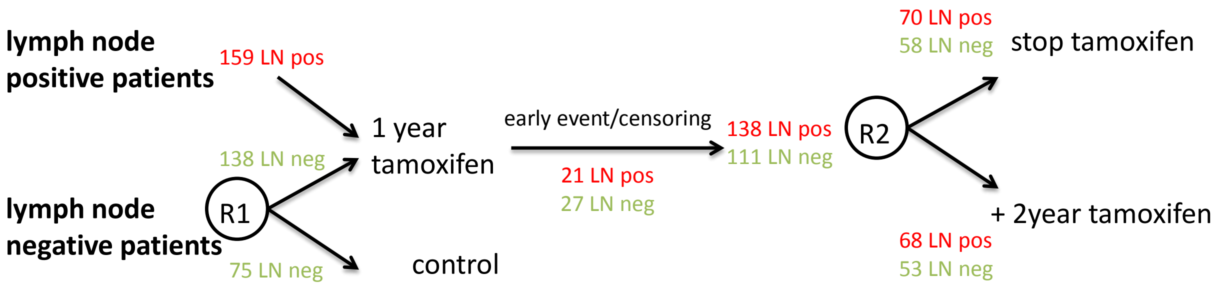

Figure S5

Numbers of patients per randomization group pre-interim analysis (A) and post-interim analysis (B), for the total subset of 563 ERα positive patients. From 1989, based on two interim analyses showing a significant improvement in recurrence-free survival among lymph node positive patients, these node positive patients were all allocated to the tamoxifen treatment arm (i.e. skipped the first randomization). Numbers of lymph node negative patients are depicted in green. In red are depicted the numbers of lymph node positive patients. Abbreviations: LN neg: lymph node negative, LN pos: lymph node positive, R1: randomization 1, R2: randomization 2.

Figure S6: Kaplan Meier survival analysis according to tamoxifen treatment in patients whose tumors express low levels of p-mTOR (A) and patients whose tumors express high p-mTOR (B).

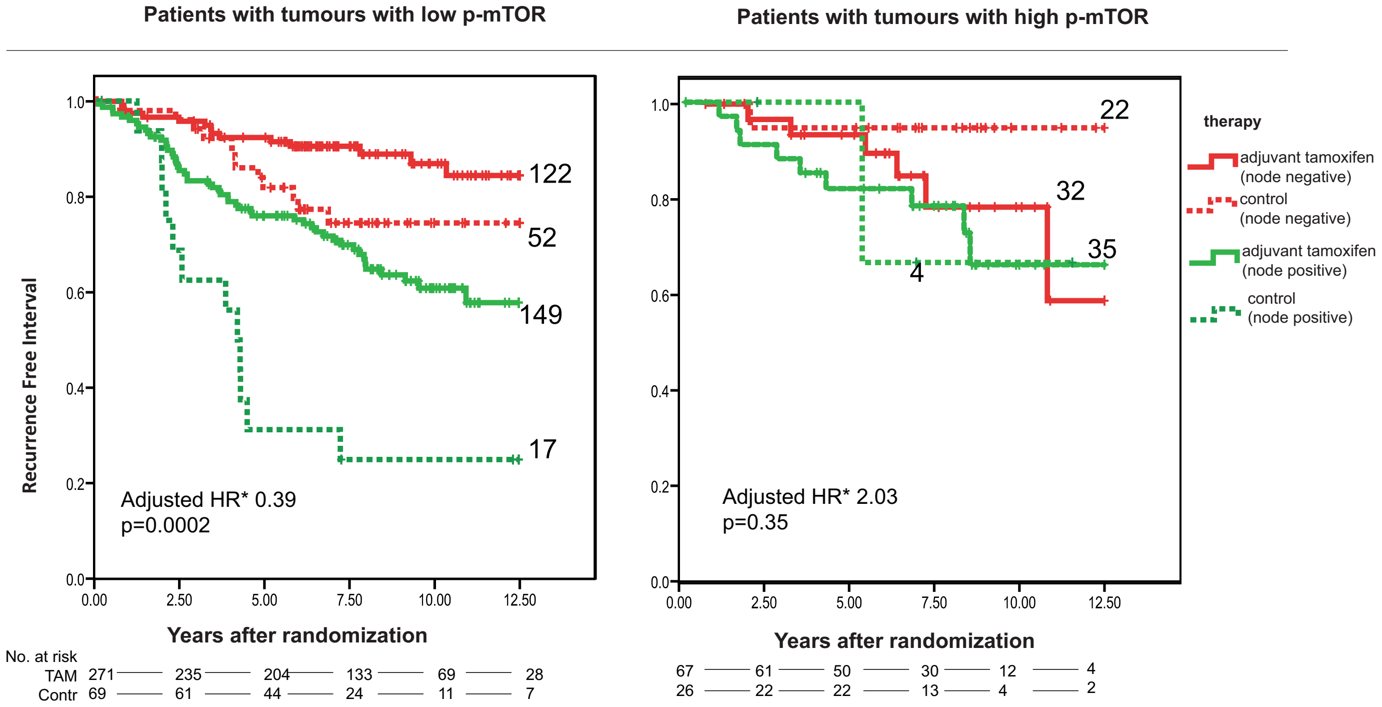

* HR stratified for nodal status

Figure S7: Kaplan Meier survival analysis according to tamoxifen treatment in patients whose tumors do not express p-AKT(Thr308) (A) and patients whose tumors do express p-AKT(Thr308)(B)

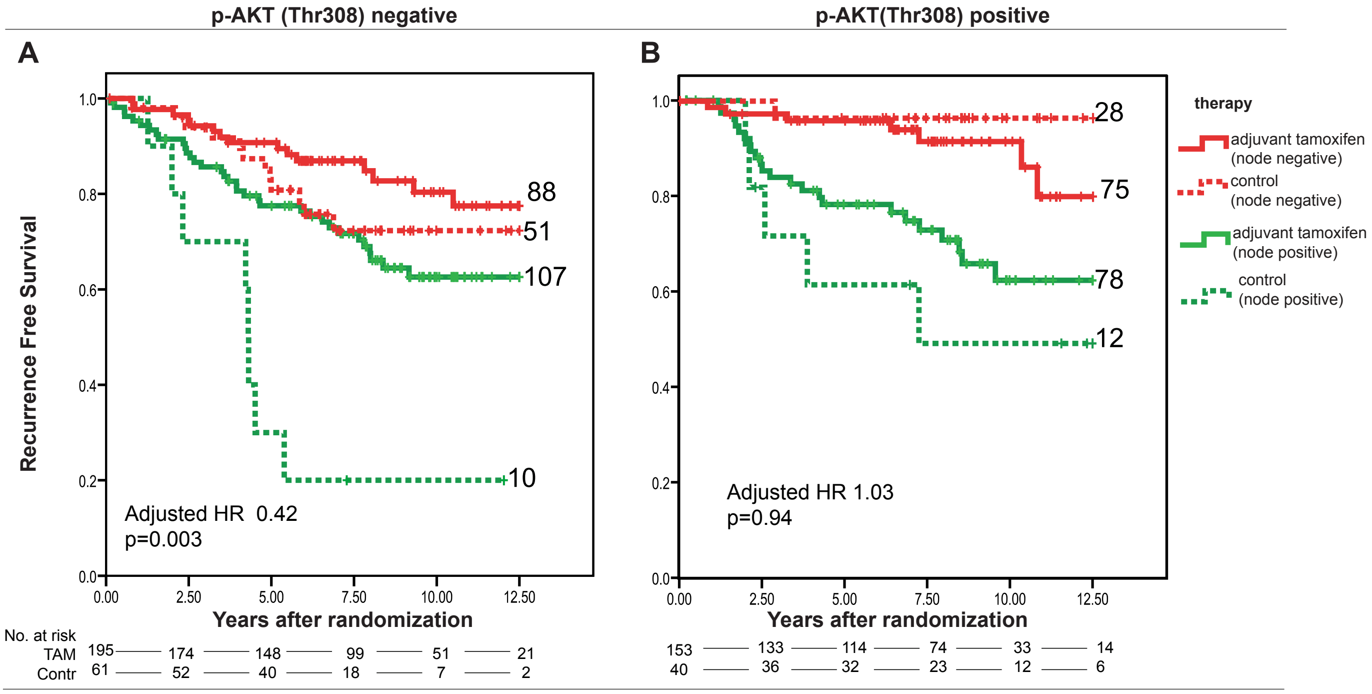

Figure S8: Kaplan Meier survival analysis according to tamoxifen treatment in patients whose tumors do not express p-ERK1/2 (A) and patients whose tumors do express p-ERK1/2 (B)

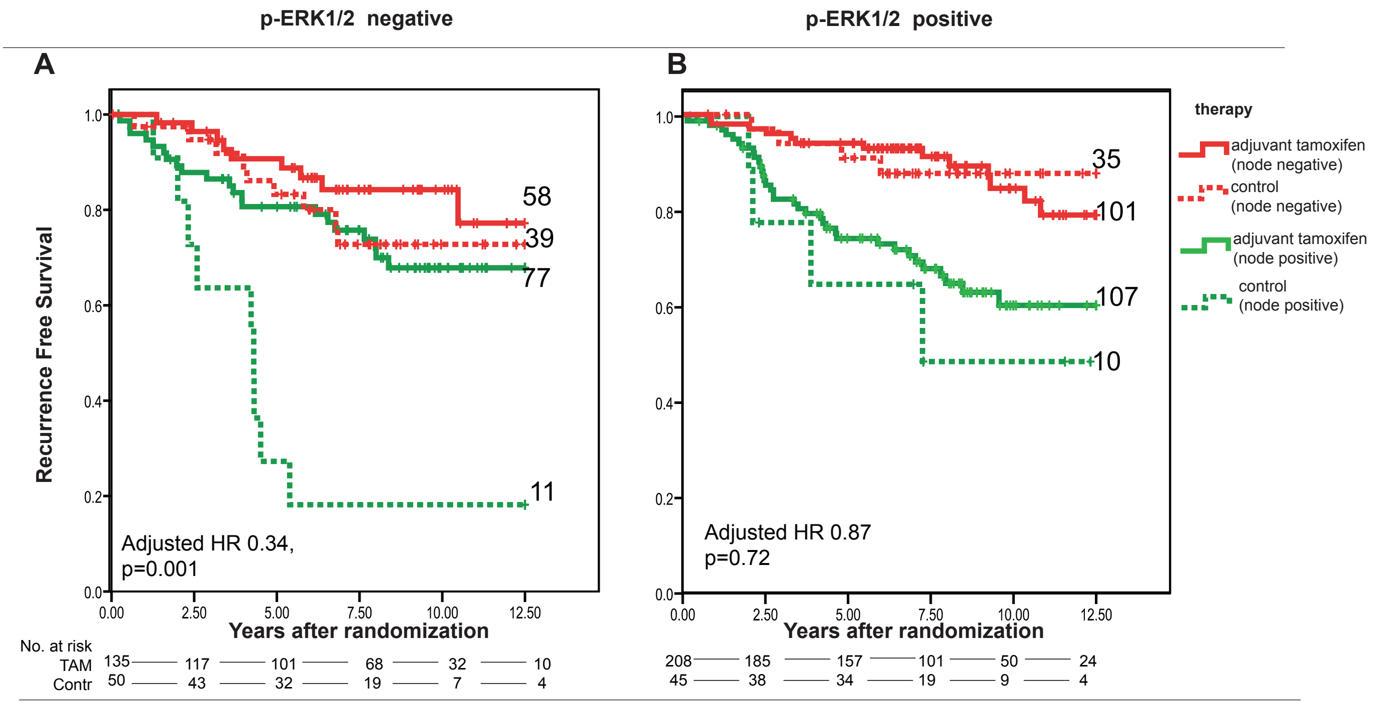

Supplement: Additional file 1 — Is Supplementary Tables S1 to S20 and Supplementary Figures S1 to S8. [file bcr3598-S1.pdf]
